# Effectiveness of telemedicine interventions on blood pressure control and self-management efficacy in hypertensive patients: a systematic review and meta-analysis

**DOI:** 10.3389/fpubh.2025.1693141

**Published:** 2026-01-12

**Authors:** Gerile Yang, Nan Zhang, Yuan Zhang, Rula Sa, Liping Zhao

**Affiliations:** 1 School of Health Management, Inner Mongolia Medical University, Hohhot, China; 2 Department of Gastroenterology, Hohhot First Hospital, Hohhot, China

**Keywords:** health behavior, hypertension, medication adherence, meta-analysis, self-management, telemedicine

## Abstract

**Objective:**

The objective of this study was to systematically evaluate the combined effect of a telemedicine intervention on systolic blood pressure (SBP), diastolic blood pressure (DBP), and self-management efficacy in adult hypertensive patients.

**Methods:**

On May 2025, a systematic literature search was conducted using the PubMed, Cochrane, Embase, and Web of Science databases. Randomized controlled trials (RCTs) were included to compare the effectiveness of the telemedicine group (the intervention group) with the usual care group (the control group) in controlling the blood pressure of adult patients with hypertension. Primary outcomes were SBP and DBP change values, and secondary outcomes included self-management, medication adherence, self-efficacy, weight, BMI, pulse pressure, total cholesterol, and low-density lipoprotein. Effect sizes were combined using a random-effects model, and heterogeneity was explored through subgroup analyses of mean age, intervention intensity, intervention duration, intervention mode, and region.

**Results:**

A total of 31 eligible articles, comprising 31 studies and 9,559 patients (4,685 in the intervention group and 4,874 in the control group), were included in the evidence synthesis analysis. The two groups had similar baseline characteristics for all outcomes. The pooled analysis revealed statistically significant changes in SBP and DBP, pulse pressure, and total cholesterol. Additionally, improvements in self-efficacy, self-management skills, and hypertension literacy were observed following the intervention. However, no significant differences were observed in medication adherence, weight, BMI and LDL. Subgroup analyses revealed that the intervention, which combined a remote monitoring device with an app, was more effective and had lower heterogeneity among individuals under 60 years of age in China and other East Asian regions.

**Conclusion:**

Telemedicine, which combines home blood pressure monitoring with remote support from healthcare providers, may become a promising strategy for managing hypertension. This approach can significantly reduce blood pressure levels and improve self-management efficacy in hypertensive patients.

**Systematic review registration:**

https://www.crd.york.ac.uk/PROSPERO/, identifier CRD420251083786.

## 1 Introduction

Hypertension is a major risk factor for cardiovascular disease (CVD) worldwide, and its prevalence continues to increase, particularly among adults. However, approximately two-thirds of people with hypertension worldwide have substandard blood pressure control (1–3). This undercontrol is particularly pronounced in specific populations—for example, African Americans in the United States face a significantly higher risk of uncontrolled blood pressure compared to whites, exacerbating health inequalities. This state of inadequate control has directly led to an increase in the incidence of events such as myocardial infarction and stroke, imposing a heavy burden on both individual health and the public health system (4–6). Furthermore, the long-term management of hypertension and the disease itself significantly impact patients’ quality of life, making its improvement a key objective in the comprehensive management of hypertension.

Traditional hypertension management primarily relies on drug therapy, but patient adherence remains generally low (approximately 50%), and standardized protocols struggle to accommodate the individualized needs of diverse patient populations (7). Telehealth interventions, especially remote monitoring, counselling, and education provided through digital platforms, are gaining attention as an emerging management modality in this context due to their promising potential (8).

Telemedicine leverages information and communication technology (ICT) to overcome the limitations of traditional in-person follow-ups. Empowering patients through remote blood pressure monitoring, online consultations, and digital self-management support holds promise for enhancing equity and quality in chronic disease management (9).

Although several studies have examined the effectiveness of telemedicine in managing hypertension and chronic diseases, limitations remain. First, most studies fail to differentiate among telemedicine tools (e.g., multimodal digital management models versus simple telephone follow-ups), thereby overlooking potential heterogeneity in outcomes due to differences in intervention components. Second, there is insufficient focus on patient behavioral and psychological outcomes (e.g., self-management capabilities, self-efficacy), with excessive reliance on physiological indicators. Additionally, there is a lack of in-depth analysis examining how geographical variations and demographic characteristics influence intervention effectiveness. To address these limitations, the innovation of this study lies in (1) refining the classification of research based on telemedicine’s technical characteristics and intervention intensity to evaluate the differentiated effects of various combined interventions; (2) systematically quantifying telemedicine’s impact on behavioral and psychological indicators such as patient self-management, self-efficacy, and hypertension knowledge; and (3) identifying key heterogeneous factors influencing intervention outcomes through regional and population subgroup analyses, thereby providing evidence-based support for developing precision blood pressure control strategies.

The objective of this study is to assess the impact of remote health interventions on blood pressure control, self-management efficacy, and quality of life in patients aged 18 years and older with hypertension through a meta-analysis of existing randomized controlled trials (RCTs). The research findings will provide an evidence-based foundation for clinical practice and inform the development of precision strategies for managing hypertension.

## 2 Methods

### 2.1 Study design and registration

The Preferred Reporting Items for Systematic Reviews and Meta-Analyses (PRISMA) standards and the Cochrane Collaboration Manual were followed in the conduct of this review (10, 11). The pre-registration protocol (registration number: CRD420251083786) was properly registered in PROSPERO.

### 2.2 Search strategy

We used four databases, PubMed, Web of Science, Embase, and Cochrane Library, to conduct a systematic literature search from the time the databases were created to May 24, 2025. We searched different databases using a combination of MeSH terms, subject terms, free terms, and Emtree. The following terms were used: “hypertension,” “self-management,” and “telemedicine.” Additionally, a comprehensive review of relevant previous systematic reviews was conducted, with the search limited to English-language publications. Detailed search strategies were provided in Supplementary Table 1. Furthermore, a manual review of the reference lists of all eligible studies was conducted. Two researchers independently retrieved and assessed applicable studies, and any discrepancies in the literature search were resolved by consensus with a third researcher.

### 2.3 Inclusion and exclusion criteria

Eligibility criteria were developed according to the PICOS framework (Population, Intervention, Comparator, Outcomes, and Study design).

#### 2.3.1 Inclusion criteria

(1) Participants: Adults aged 18 years and older diagnosed with hypertension, regardless of gender, ethnicity, or socioeconomic status. Participants were required to have a baseline systolic blood pressure (SBP) of 130 mmHg or higher or diastolic blood pressure (DBP) of 80 mmHg or higher (12).
(2) Interventions: Telehealth interventions aimed at blood pressure management, including but not limited to remote monitoring, mobile health applications, and educational programs. Interventions were required to be delivered via digital platforms (e.g., video calls, phone calls, or mobile apps).
(3) Comparator: Control groups receiving usual care for hypertension management.
(4) Outcomes: Primary outcomes were required to include changes in blood pressure (measured as SBP and DBP) from baseline to follow-up. Secondary outcomes included self-management, medication adherence, self-efficacy, weight, BMI, total cholesterol, and low-density lipoprotein.
(5) Study design: This systematic review exclusively encompassed randomized controlled trial (RCT) studies that were published in the English language.

#### 2.3.2 Exclusion criteria

(1) Non-randomized controlled trials, non-English publications, or publication types for which the full text is unavailable (e.g., conference abstracts, academic theses).
(2) Studies included participants younger than 18 years of age or with secondary hypertension or significant comorbidities that could impact blood pressure management.
(3) Non-telehealth interventions, or studies that lacked a control group receiving usual care only.
(4) Studies did not include a control group that did not receive telemedicine intervention.
(5) Duplicate publications (with priority given to the study report with the largest sample size and most comprehensive data).

### 2.4 Data extraction

The data extraction process was executed independently by two researchers, employing standardized tables within the Microsoft Excel (2021) software, with a third researcher explaining and making the final decision in case of any disagreement. The following data were extracted from the studies that were ultimately included: (1) trial characteristics, including the first author, year of publication, study country, sample size, and study duration; (2) patient characteristics, including age, sex, weight, BMI, systolic pressure, diastolic pressure, self-management, medication adherence, self-efficacy, total cholesterol, and low-density lipoprotein; (3) characteristics of telemedicine interventions. Additionally, we employed mathematical techniques to translate the studies’ continuous variables into mean ± standard deviation, which have been proved (13). For studies where data were missing or unobtainable, we attempted to contact the authors to obtain the data.

### 2.5 Assessment of study quality and risk of bias

Two researchers independently evaluated the quality and level of evidence of eligible studies using the Cochrane Collaboration’s Risk of Bias Assessment Tool. Any divergences were resolved through discussion or by consulting a third reviewer. The tool evaluated studies based on seven core dimensions to determine their methodological quality: (1) the process of randomization sequence generation, (2) the concealment of allocation, (3) the blinding of participants and researchers, (4) the blinding of during outcome assessment, (5) the completeness of outcome data and handling of missing data, (6) selective reporting of study results, and (7) the potential for other sources of bias. Each domain was rated as having a ‘low risk’, ‘high risk’, or ‘unclear risk’ of bias. The summary analysis of bias risk was performed using Review Manager 5.4 software (Cochrane Collaboration, Oxford, United Kingdom).

### 2.6 Data analysis

This study used Review Manager 5.4 to perform a meta-analysis. Continuous data were synthesized using the mean difference (MD) ± SD to evaluate the intervention’s effectiveness and calculate the effect size. The *I*^2^ index was used to assess study heterogeneity. When heterogeneity was not significant (*p* ≥ 0.05 and *I*^2^ ≤ 50%), a fixed-effects model was used; when significant heterogeneity was present (*p* < 0.05 or *I*^2^ > 50%), a random-effects model was employed. We conducted sensitivity analyses to assess the robustness of the pooled results by sequentially excluding individual studies. We used Review Manager 5.4 to create funnel plots in order to visually measure publication bias. Finally, we performed subgroup analyses to determine the effectiveness of specific telemedicine interventions in reducing SBP and DBP and other factors based on population characteristics and intervention measures.

## 3 Results

### 3.1 Literature screening process

**Figure 1:**
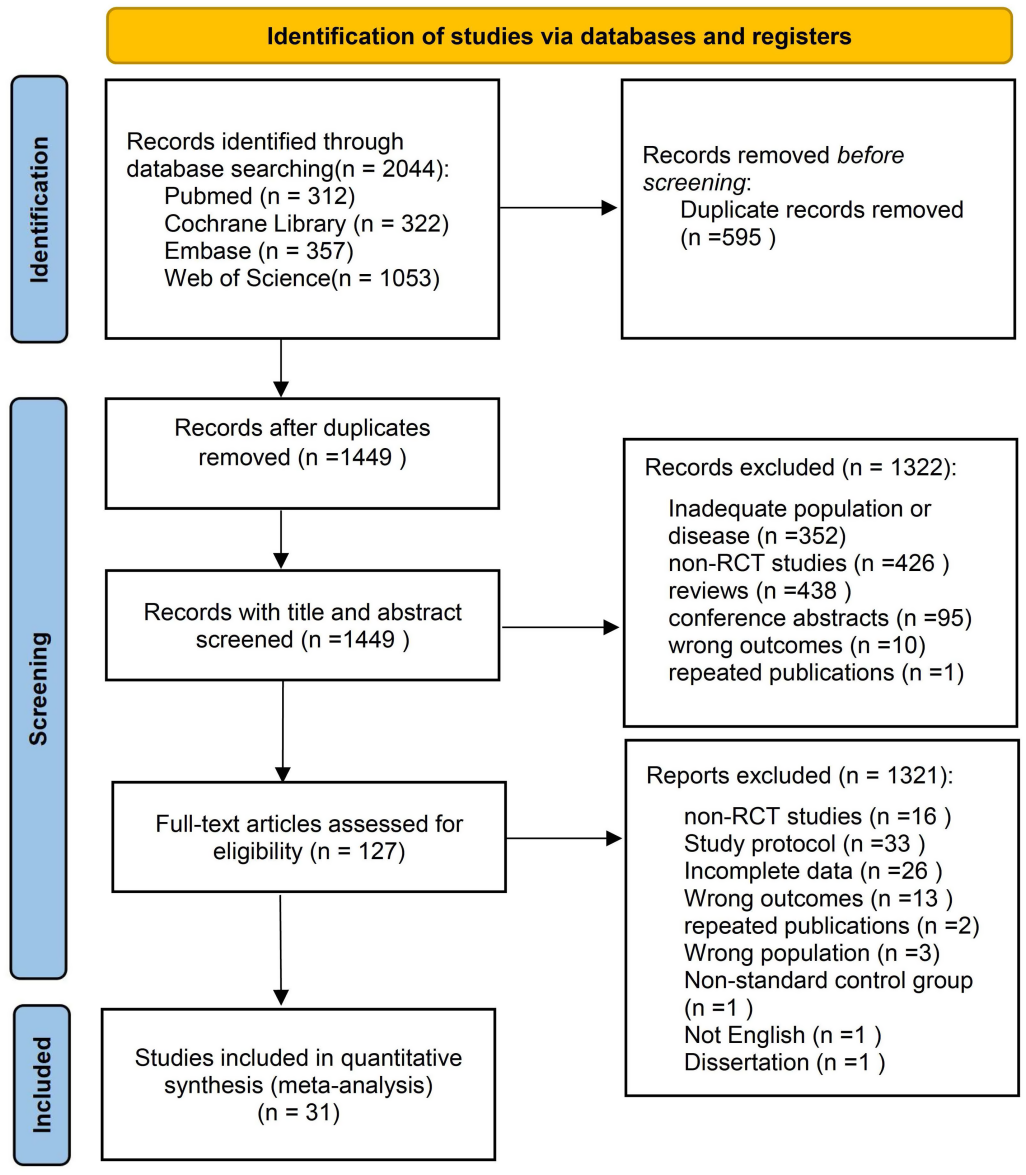
Flowchart of literature selection.

Through a systematic literature search, we obtained a total of 2,044 relevant publications from PubMed (*n* = 312), Cochrane (*n* = 322), Embase (*n* = 357), and Web of Science (*n* = 1,053) databases. After removing duplicates, we screened 1,449 titles and abstracts. Ultimately, 31 full-text articles covering 31 studies with a total of 9,559 patients (4,685 in the intervention group and 4,874 in the control group) were included in the comprehensive analysis. Figure 1 shows a schematic of the systematic search and screening process. Supplementary Table 2 details the full-text articles excluded during the systematic review screening process and the rationale for their exclusion.

### 3.2 Study characteristics

Of these studies, eight were from China (14–21), eight were from the United States (22–29), four were from the United Kingdom (30–33), three were from South Korea (34–36), two were from Canada (37, 38), and one each was from Japan (39), Turkey (40), Jordan (41), Spain (42), Germany (43), and Peru (44). The publication dates ranged from 2010 to 2025 (Table 1). A total of 16 articles were published in the last 5 years, which is a relatively average number of articles per year. The number of articles published from 2020 to 2025 was three, two, two, two, and two, respectively. The intervention durations ranged from 1.5 to 12 months. Nine studies lasted 12 months, 10 lasted 6 months, and seven lasted 3 months. Telemedicine intervention methods encompass a range of approaches, including telephone, text messaging, remote monitoring devices, applications, and combinations of remote monitoring devices and applications or telephone. Remote monitoring devices included home blood pressure monitoring devices, sensors, and wearable patches. Apps were mostly developed by healthcare professionals or existing apps, such as WeChat. The intervention process involves patients filling in basic information, such as blood pressure, medication, diet, and exercise, in the app. Healthcare professionals then provide professional hypertension education based on this information.

**Table 1:** Characteristics of included studies.

Author	Year	Country	Type	Number of subject (intervention/control)	Average age	Intervention introduction	Duration	Outcome measure
Li et al.	2019	China	RCT	110/143	61.5 ± 6.4	WeChat official account health articles (3 months) + check-in system + weekly group discussions (low-medium risk 1 time/week, high risk 2 times/week) + private coaching consultation + blood pressure self-monitoring feedback	6 months	SBP, DBP, hypertension knowledge, self-efficacy, self-management score
Karen et al.	2022	America	RCT	1423/1648	60.2 ± 14.4 Intervention: 62.4 ± 14.2 Control: 58.3 ± 14.2	Remote follow-up: telephone every 2–4 weeks, based on home blood pressure adjustment therapy. Additional: face-to-face initial assessment by pharmacist (1 h) and home blood pressure monitoring equipment to remote monitoring of family blood pressure.	4 months	SBP, DBP
Sun et al.	2024	China	RCT	23/31	67.24 ± 4.19 Intervention: 67.09 ± 4.43 Control: 67.32 ± 4.06	The intelligent health cabin is used to complete the test and generate personalized reports, issue personalized exercise prescriptions and simplify DASH dietary guidance. WeChat intervention: Text messages based on behavior modification technology are pushed weekly with dynamic feedback on exercise, diet and medication effects.	3 months	SBP, DBP, medication adherence, weight
Tam et al.	2023	China	RCT	35/34	67.39 ± 9.98 Intervention: 66.82 ± 10.5 Control: 67.97 ± 9.5	1. One face-to-face group health education. 2. Tailored leaflet with QR codes: The leaflet summarized the hypertension-related knowledge, and quick response (QR) codes to watch the exercise video. 3. Weekly text messaging.	3 months	SBP, DBP, medication adherence, pulse pressure, self-efficacy
Karen et al.	2013	America	RCT	197/191	61.1 ± 12.0 Intervention: 62.0 ± 11.7 Control: 60.2 ± 12.2	Home blood pressure remote monitoring + pharmacist follow-up	12 months	SBP, DBP
Frias et al.	2017	America	RCT	39/27	58.7 Intervention: 56.7 ± 11.24 Control: 61.6 ± 8.83	Acceptance of digital medicine offering (DMO) 1. Ingestible sensors + drug encapsulation; 2. Wearable patches monitor activity, heart rate, and steps, with data recorded by a mobile app. Doctors adjust medication based on DMO data.	3 months	SBP, DBP, TC, LDL-C
Liu et al.	2018	Canada	RCT	39/39	56.9 ± 7.07 Intervention: 57.6 ± 9.37 Control: 56.1 ± 8.74	Based on Canadian physical activity guidelines and dietary guidelines, experts pre-set exercise and diet plans and send users electronic newsletters on hypertension management.	4 months	SBP, DBP, pulse pressure, TC
Thiboutot et al.	2013	America	RCT	282/218	60.5 ± 11.9 Intervention: 59.6 ± 12.1 Control: 61.6 ± 11.4	Patients use a fully automated website to receive monthly customized hypertension management questions (based on JNC 7 guidelines).	12 months	SBP, DBP
Kim	2019	Korea	RCT	31/31	77.70 ± 6.92 Intervention: 78.25 ± 6.61 Control: 77.70 ± 6.92	Telephone health guidance based on Cox’s Interaction Model of Client Health Behavior (IMCHB); customized long-message services (LMS)	2 months	SBP, DBP, medication adherence, hypertension knowledge, self-efficacy, self-management score
Wang et al.	2023	China	RCT	88/87	50.8 ± 14.2 Intervention: 50.9 Control: 50.7	Multimodal digital management model: based on WeChat platform, integrating three core modules of remote monitoring, personalized education and doctor–patient interaction.	6 months	SBP, DBP
Liu et al.	2023	China	RCT	111/115	49.6 Intervention: 48.58 ± 9.54 Control: 50.64 ± 8.72	Using the “Blood Pressure Assistant” App, which includes health monitoring, education and reminder modules, patients upload data every day, and medical teams intervene remotely based on abnormal values.	6 months	SBP, DBP, hypertension knowledge, BMI
McManus et al.	2018	UK	RCT	328/348	66.9 ± 9.4 Intervention: 67.0 Control: 66.8	Based on the automatic upload of blood pressure data from home blood pressure self-monitoring (SMBP), the system generates trend charts and gives early warnings. The GP adjusts the medication according to the target of home blood pressure control.	12 months	SBP, DBP
Yatabe et al.	2021	Japan	RCT	48/46	53 ± 9	The telemedicine group used a 3G network–attached home blood pressure (BP) monitoring device, consulted hypertension specialists from an academic hospital through web-based video visits, and received prescription medication.	12 months	SBP, DBP
Keskin et al.	2025	Turkey	RCT	40/40	55.13 ± 11.15 Intervention: 52.2 ± 11.57 Control: 58.07 ± 10.02	Use the mobile app to record blood pressure value, medication section, exercise section, diet section, with daily reminders and feedback on trend charts.	6 weeks	SBP, DBP, medication adherence, self-management score, weight, pulse pressure, BMI
Nolan et al.	2018	Canada	RCT	100/97	57.6 ± 8.42 Intervention: 57.2 Control: 58.0	The e-counseling intervention is based on motivational interviewing (MI) and cognitive-behavioral therapy (CBT) to reinforced efficacy via goal setting, progressive steps, self-monitoring tools and video-based peer modeling.	12 months	SBP, DBP, pulse pressure, TC, LDL-C
Lesli et al.	2018	America	RCT	41/32	58	Automated BP monitoring, tailored feedback texts and stratified health texts.	6 months	SBP, DBP
Alsaqer et al	2022	Jordan	RCT	37/37	Intervention: 60.37 ± 5.60 Control: 60.37 ± 5.60	Telephone follow-up once a week (10 min) for 3 months, combined with WhatsApp group support. Nurses guide self-care based on tertiary prevention, including assessing patient knowledge/behavior, checking application data, providing feedback and guidance, and encouraging interaction among patients.	3 months	SBP, DBP
Márquez et al.	2018	Spain	RCT	73/75	57.5 ± 9.9 Intervention: 57.7 ± 9 Control: 57.08 ± 10	Special APP “ALERHTA” and educate people about hypertension health knowledge.	12 months	SBP, DBP
Antonio et al.	2020	Peru	RCT	80/84	30–60 Average age not directly reported	Have a motivational interview call once a month, input the phone information into the network platform, and send customized SMS every week.	12 months	SBP, DBP, weight, BMI
McManus et al.	2010	UK	RCT	234/246	35–85 Average age not directly reported	Patients measure their blood pressure and upload the data through a secure website/telephone, and nurses monitor them remotely.	12 months	SBP, DBP
Sun et al.	2020	China	RCT	59/58	Intervention: 52.35 ± 9.46 Control: 53.42 ± 8.78	According to cardiovascular risk stratification (low, medium and high risk groups), three WeChat groups were established to push differentiated content (low-medium risk group once a week, high risk group twice a week). The content of WeChat intervention includes health education, behavior promotion and blood pressure monitoring; adjust the dosage and time of medication according to blood pressure fluctuations; and support WeChat consultation.	3 months	SBP, DBP, medication adherence, TC, LDL-C, BMI, self-management score
Kim et al.	2016	America	RCT	52/43	57.6 Intervention: 57.5 ± 8.6 Control: 57.7 ± 8.7	Withings blood pressure monitor is equipped with wireless connection to the mobile phone and automatically uploads data to the HealthyCircles platform. It provides iPhone and supporting App, including educational materials and reminder function. Nurses can monitor data through the platform and provide behavioral suggestions (such as diet and exercise), but do not directly adjust drugs.	6 months	SBP, DBP, medication adherence
Yoon et al.	2024	Korea	RCT	85/88	59.8 ± 11.6 Intervention: 60.3 ± 11.2 Control: 59.3 ± 16.1	Using the Bluetooth sphygmomanometer, the data can be automatically uploaded through the mobile application. The built-in algorithm can provide real-time suggestions according to the blood pressure value, and the doctor can monitor the real-time (eCRF Lite system). The nurse can observe the abnormality and contact the patient through the platform.	6 months	SBP, DBP
Ye et al.	2024	China	RCT	78/77	57.6 ± 4.5 Intervention: 57 ± 3.8 Control: 58.2 ± 5.1	WeChat platform intervenes to establish a WeChat group, push disease knowledge and management norms every day, answer questions in real time, and give personalized guidance.	26 weeks	SBP, DBP, weight, BMI, TC, LDL-C
Calvin et al.	2016	China	RCT	33/28	69.5 ± 9.9 Intervention: 69.3 ± 9.7 Control: 69.7 ± 10.2	Use a blood pressure monitor to measure blood pressure and automatically remind when the measured value deviates from the normal range; video education includes chronic disease management content such as measurement operation, diet, and exercise.	3 months	SBP, DBP
Kim et al.	2015	Korea	RCT	126/124	58.8 ± 10.6 Intervention: 56.1 ± 11 Control: 56.4 ± 9.9	LG Smart Care system + outpatient follow-up, doctors adjust medication based on remote data.	6 months	SBP, DBP
McKinstry et al.	2013	United Kingdom	RCT	182/177	Intervention: 60.5 ± 11.8 Control: 60.8 ± 10.7	Using the Stabil-O-Graph wireless blood pressure monitor to measure blood pressure, the data is automatically uploaded to a secure website via Bluetooth and feedback on blood pressure management is sent by SMS or email. The general practitioner (GP) or nurse checks the website data weekly and adjusts medication according to guidelines.	6 months	SBP, DBP, self-efficacy, BMI, TC
McManus et al.	2021	United Kingdom	RCT	305/305	Intervention: 65.2 ± 10.3 Control: 66.7 ± 10.2	Use a smart blood pressure monitor to measure blood pressure, automatically upload data to a secure platform, and receive feedback via a built-in traffic light system (green: within normal range, yellow: needs attention, red: needs intervention). Doctors can preset a three-level medication adjustment plan (triggered if blood pressure is not within normal range for 2 consecutive months) and provide behavioral support.	12 months	SBP, DBP
Meyer et al.	2025	Germany	RCT	52/50	54.5 ± 8.0 Intervention: 54.9 Control: 54.1	The internet-based digital therapy, Liebria, combines cognitive behavioral therapy (CBT) and lifestyle change counseling.	3 months	SBP, DBP, pulse pressure, medication adherence
Persell et al.	2020	America	RCT	144/152	58.9 ± 12.8 Intervention: 59.6 Control: 58.3	The AI-powered hypertension management app combines cognitive behavioral therapy with personalized monitoring of blood pressure, medication reminders, dietary guidance, exercise plans, sleep optimization, and stress management. Through conversational AI, it delivers tailored recommendations, automatically syncs blood pressure data, alerts users to abnormal readings, and suggests medical consultations when needed.	6 months	SBP, DBP, self-efficacy, BMI
Prendergast et al.	2025	America	RCT	210/203	51.1 ± 12.5 Intervention: 50.3 Control: 51.9	Provides Bluetooth sphygmomanometer and Health Mate app, supports daily self-test, automatic data synchronization, weekly SMS reminders to promote medication compliance and self-monitoring, and clinical pharmacist or advanced practice nurse (APN) provides hypertension education.	6 months	SBP, DBP

SBP, systolic blood pressure; DBP, diastolic blood pressure; BMI, Body Mass Index; TC, total cholesterol; LDL-C, low-density lipoprotein cholesterol; RCT, randomized controlled trial.

### 3.3 Risk of bias

**Figure 2:**
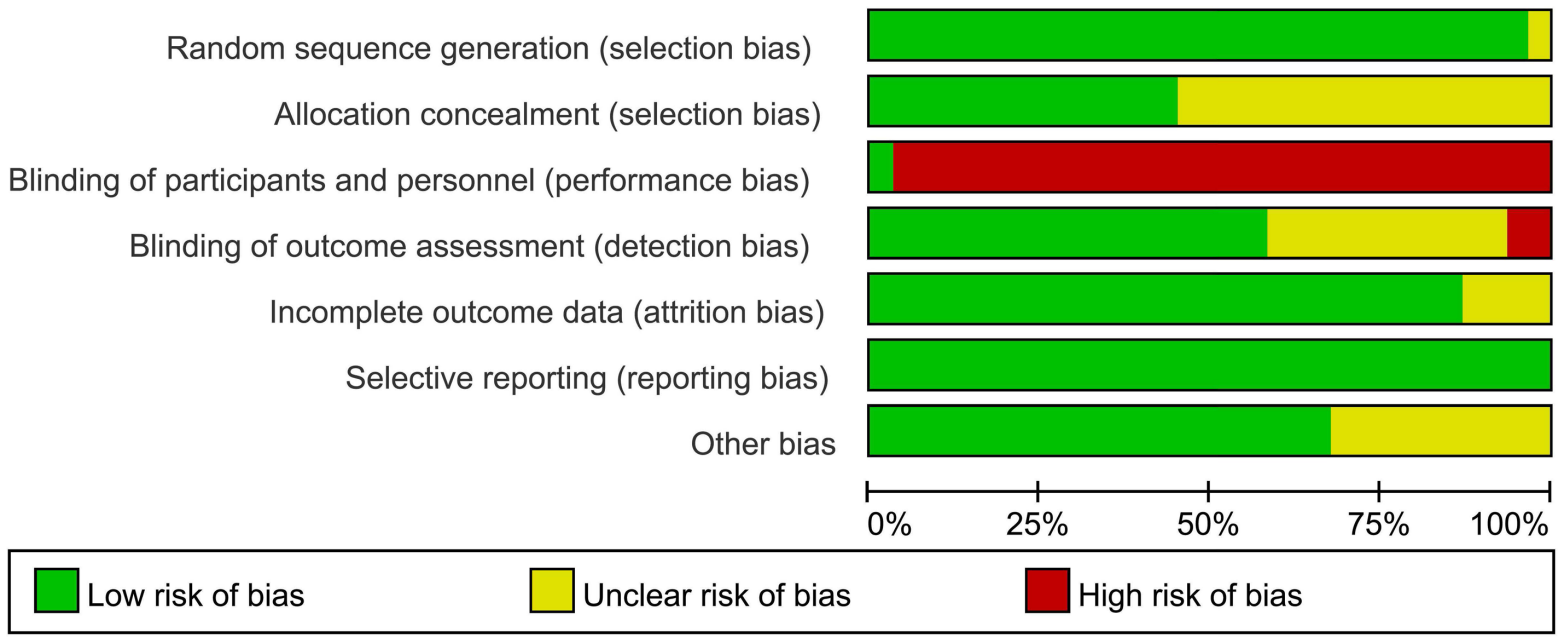
Results of the risk of bias analysis.

**Figure 3:**
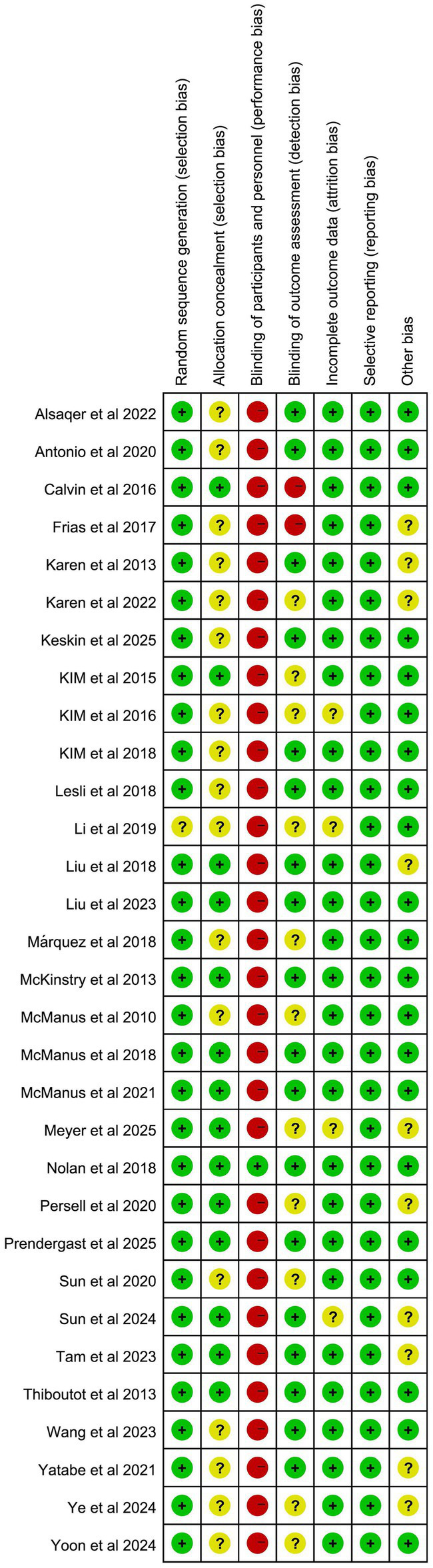
Results of the risk of bias analysis.

Of the literature included in the final analysis, randomized sequences generated 30 low-risk ratings and one unclear rating. Due to the lack of apparent allocation concealment, 17 studies were rated as unclear, while the remaining 14 studies were rated as having a low risk of bias. Due to the specific nature of telemedicine interventions, most studies did not employ blinding for interveners, subjects, or outcome measures. Thus, 30 articles were rated as high risk, while only one was rated as low risk. Regarding blinding of outcome assessors, two articles were rated as high risk, 11 as unclear, and 18 as low risk. In terms of the completeness of the outcome data, 27 articles were rated as low risk because telemedicine communication was easy. An additional four articles were rated as unclear. All literature was low risk in terms of selective reporting. Ten articles were rated as unclear and 21 as low risk for other biases. The results of the detailed risk of bias assessment are shown in Figures 2, 3.

### 3.4 Results of the meta-analysis

#### 3.4.1 Effect on systolic blood pressure (SBP)

A meta-analysis of 31 studies (intervention group *n* = 4,685, control group *n* = 4,874) demonstrated that telemedicine interventions significantly reduced systolic blood pressure (MD: −4.62; 95% CI: −5.78, −3.46; *z*-value: 7.8; *p* < 0.001; *p* = 0.01; *I*^2^ = 40%, random-effects model). This magnitude of reduction is consistent with guidelines that associate a 2–5 mmHg decrease in population-level SBP with a meaningful 5–10% lower risk of major cardiovascular events (45). Moderate heterogeneity was observed in the results. Sensitivity analysis indicated that the study by Karen et al. was the primary source of heterogeneity, which disappeared after exclusion.

#### 3.4.2 Effect on diastolic blood pressure (DBP)

31 studies investigated the effect of telemedicine on DBP (intervention group *n* = 4,685, control group *n* = 4,874). Meta-analysis results indicated that telemedicine intervention also significantly reduced diastolic blood pressure (MD: −1.33; 95% CI: −1.91, −0.76; *z*-value = 4.53, *p* < 0.001; *p* = 0.18, *I*^2^ = 18%, fixed-effects model). No significant heterogeneity was observed, and the funnel plot did not indicate publication bias.

#### 3.4.3 Effect on medication adherence

A meta-analysis of seven studies (intervention group *n* = 292, control group *n* = 287) indicated a non-significant improvement in medication adherence favoring the telemedicine group (SMD: 0.25; 95% CI: −0.03, 0.54; *z*-value 1.73, *p* = 0.08; *p* = 0.008, *I*^2^ = 65%, random-effects model), with considerable heterogeneity and no evidence of publication bias. The high heterogeneity, largely attributable to the studies by Kim et al. (27, 34, 36) and Sun et al. (15, 19) as shown in sensitivity analysis, suggests that the effectiveness of telemedicine may vary significantly across different intervention designs and patient populations.

#### 3.4.4 Effect on hypertension knowledge score

Three studies reported on the impact of telemedicine on hypertension knowledge scores (intervention group *n* = 252, control group *n* = 289). The results demonstrated that the intervention group achieved a statistically significant improvement in hypertension knowledge compared to the control group (SMD: 0.31; 95% CI: 0.14, 0.48; *z*-value 3.61, *p* < 0.003; *p* = 0.4, *I*^2^ = 0%, fixed-effects model), with no significant heterogeneity. This finding holds significant clinical value. A visual assessment of the funnel plot revealed no evidence of publication bias.

#### 3.4.5 Effect on self-efficacy

Five studies reported on the impact of telemedicine on self-efficacy (intervention group *n* = 502, control group *n* = 537). Telemedicine significantly increased self-efficacy levels in the intervention group (SMD: 0.16; 95% CI: 0.04, 0.29; *z*-value 2.64, *p* = 0.008; *p* = 0.34, *I*^2^ = 12%, fixed-effects model), with no significant heterogeneity. A visual assessment of the funnel plot revealed no evidence of publication bias.

#### 3.4.6 Effect on self-management

Four studies reported on the impact of telemedicine on self-management (intervention group *n* = 240, control group *n* = 272). Telemedicine significantly improved self-management levels in the intervention group (SMD: 0.59; 95% CI: 0.28, 0.89; *z*-value 3.77, *p* < 0.001; *p* = 0.06, *I*^2^ = 60%, random-effects model), but with high heterogeneity. Sensitivity analysis demonstrated that the overall outcomes of self-management levels exhibited instability, attributable to the study by Sun et al. (15, 19). The exclusion of this study led to the dissolution of heterogeneity (*p* = 0.26; *I*^2^ = 25%).

#### 3.4.7 Effect on weight

Four studies reported on the impact of telemedicine on weight (intervention group *n* = 221, control group *n* = 232). The results indicated that the intervention group experienced a reduction in body weight compared to the control group (MD: −1.39; 95% CI: −2.11, −0.67; *z*-value 3.77, *p* = 0.0002; *p* = 0.99, *I*^2^ = 0%, random-effects model). However, sensitivity analysis revealed that the finding was unstable and highly dependent on a single study Ye et al. (20). Upon excluding this study from the analysis (MD: −0.62; 95% CI: −6.80, 5.56; *z*-value 0.20, *p* = 0.84; *p* = 0.98, *I*^2^ = 0%), which did not reveal a statistically significant difference.

#### 3.4.8 Effect on pulse pressure

Five studies reported on the impact of telemedicine on pulse pressure (intervention group *n* = 266, control group *n* = 260). The results indicated that pulse pressure was significantly lower in the intervention group compared to the control group (MD: −3.26; 95% CI: −4.99, −1.53; *z*-value: 3.70; *p* < 0.001; *p* = 0.68; *I*^2^ = 0%; fixed-effects model), with no significant heterogeneity observed. Additionally, a visual assessment of the funnel plot did not reveal any apparent publication bias.

#### 3.4.9 Effect on BMI

Seven studies reported on the impact of telemedicine on body mass index (BMI) (intervention group *n* = 694, control group *n* = 703). The results indicated a significant reduction in BMI within the intervention group compared to the control group (MD: −0.40; 95% CI: −0.58, −0.23; *z*-value: 4.50, *p* < 0.001; fixed-effects model), no heterogeneity (*I*^2^ = 0%). A visual examination of the funnel plot showed no evidence of publication bias. Sensitivity analysis indicated that the overall results for BMI were unstable due to the influence of Ye et al. (20); however, even after excluding this study, there was no statistically significant heterogeneity (*I*^2^ = 0%), and no statistically significant difference was found (*z*-value: 1.08, *p* = 0.28). This may be related to the fact that studies by Ye et al. (20) used more intensive intervention models. These included daily interactions and supervision via WeChat by multidisciplinary teams. Sensitivity analyses indicate that routine or lower-intensity telemedicine interventions may have limited effects on BMI.

#### 3.4.10 Effect on total cholesterol (TC)

Six studies reported on the impact of telemedicine on total cholesterol (TC) (intervention group *n* = 497, control group *n* = 475). The main meta-analysis demonstrated a statistically significant reduction in the intervention group (MD: −0.31; 95% CI: −0.58 to −0.05; *z*-value: 2.30, *p* = 0.02; *p* = 0.01, *I*^2^ = 65%, random-effects model) however exhibited high heterogeneity. A visual assessment of the funnel plot indicated no evidence of publication bias. The sensitivity analysis showed that the overall results for TC were unstable due to the studies by Ye et al. (20) and Liu et al. (18, 37). Excluding these studies resulted in the removal of heterogeneity (*p* = 0.94; *I*^2^ = 0%), yet no statistically significant difference was observed (*z*-value: 0.62, *p* = 0.54). These findings suggest that the effectiveness of telemedicine interventions in reducing TC levels is inconsistent and unstable across different studies, indicating that their clinical significance may be limited.

#### 3.4.11 Effect on low-density lipoprotein (LDL)

Four studies reported on the impact of telemedicine on low-density lipoprotein (LDL) (intervention group *n* = 276, control group *n* = 259). The results showed that LDL levels were lower in the intervention group than in the control group (MD: −0.39; 95% CI: −1.03, 0.24; *z*-value: 1.21, *p* = 0.23; *p* < 0.001, *I*^2^ = 91%, random-effects model), with high heterogeneity. Although sensitivity analysis indicated that this result was unstable and became non-significant upon removal of an influential study by Ye et al. (20), the consistent direction of effect across studies is clinically noteworthy.

**Figure 4:**
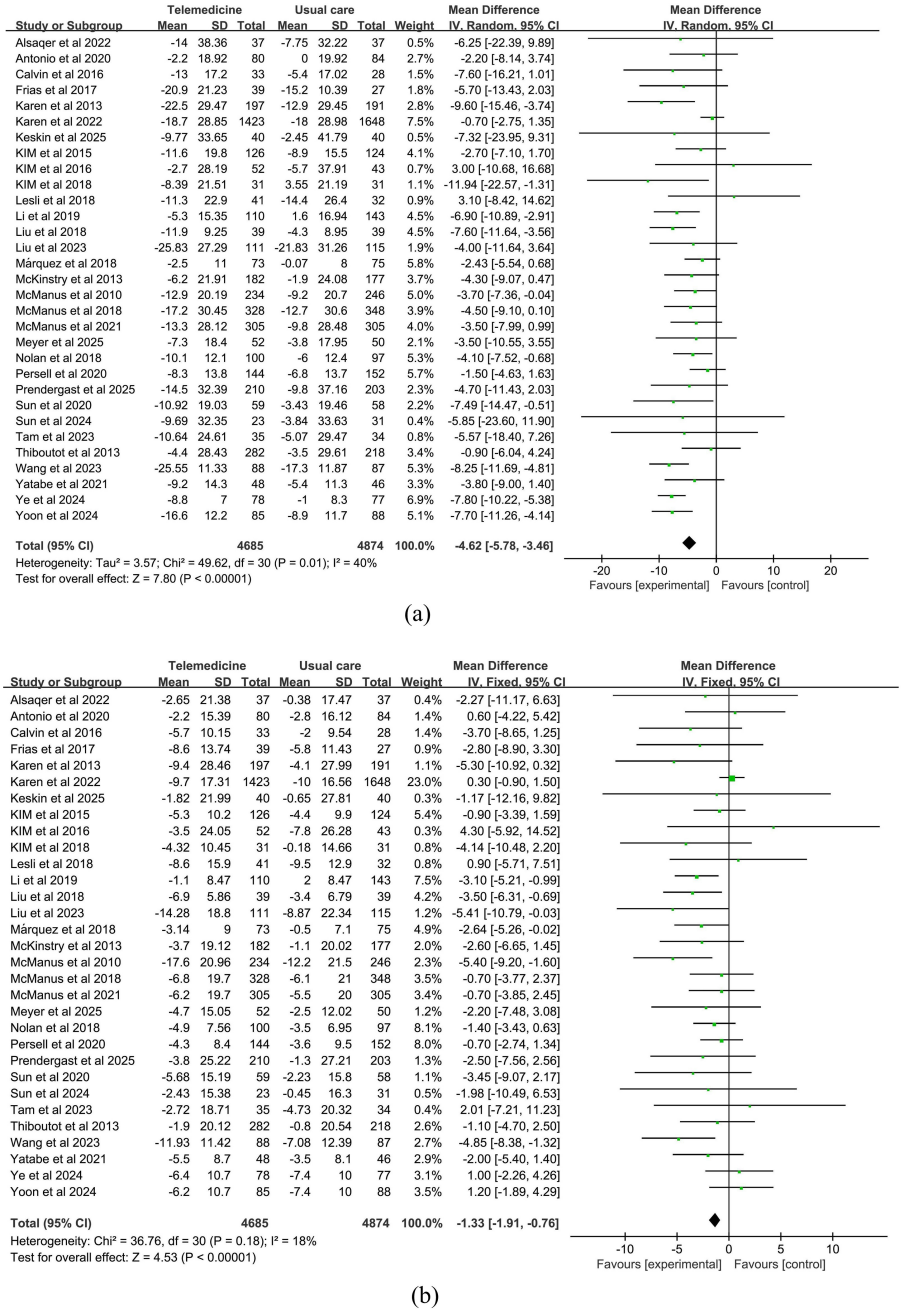
Forest plot. **(a)** Effect of systolic blood pressure (SBP). **(b)** Effect on diastolic blood pressure (DBP).

Forest plots for SBP and DBP are shown in Figure 4. Forest Plots for the other various outcome measures in Supplementary Figure 1. Funnel plots for all outcome measures are provided in Supplementary Figure 2.

### 3.5 Sensitivity analyses

We employed a stepwise exclusion method for sensitivity analysis to assess the impact of each study on the pooled effect size and heterogeneity. The results indicate that the robustness of the primary findings in this study varies. For outcomes such as systolic blood pressure and self-management, the pooled results showed high dependence, with the primary sources of heterogeneity identified as the studies by Karen et al. and Sun et al. (15, 19). After excluding these studies, the heterogeneity significantly decreased or disappeared. It is particularly noteworthy that the significant benefits in weight, TC, and LDL disappeared after excluding the studies by Ye et al. (20) and Liu et al. (18, 37), indicating that these results are unstable. In contrast, pooled estimates for outcomes such as diastolic blood pressure, hypertension knowledge, self-efficacy, pulse pressure, and BMI remained stable across sensitivity analyses, with consistently low levels of heterogeneity, demonstrating the robustness of these findings.

### 3.6 Subgroup analyses

Subgroup analyses were then conducted to explore the effects of various factors on SBP and DBP in telemedicine interventions. The following factors were considered in the analysis: the mode of the intervention (APP, remote monitoring devices, remote monitoring devices + telephone, and remote monitoring devices + APP), the duration of the intervention (<6 months and ≥6 months), the average age (≥60 years and <60 years), the region (China, North America, Europe, other East Asian countries, and other countries), and intervention content (education-focused, monitoring and feedback-focused, and comprehensive support-focused).

#### 3.6.1 Intervention mode

**Figure 5:**
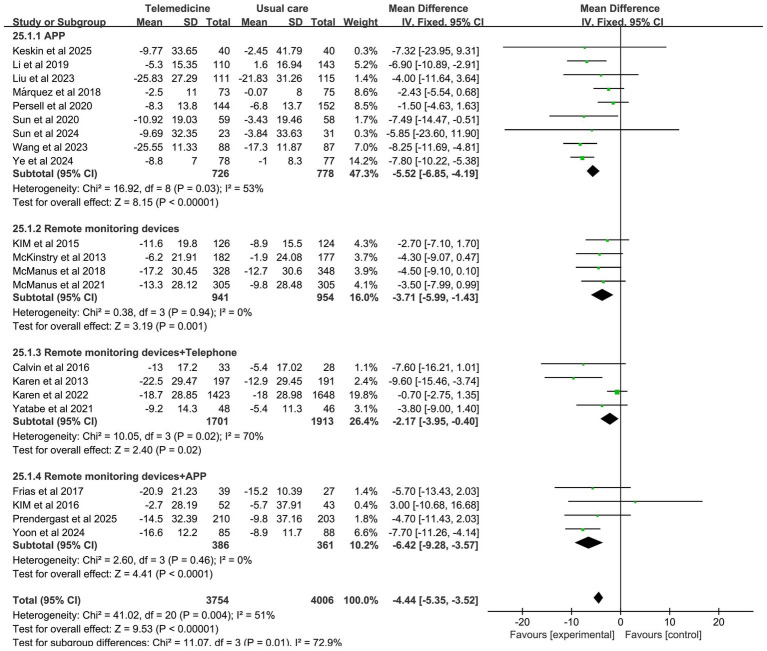
Subgroup analysis of intervention effects on SBP.

**Figure 6:**
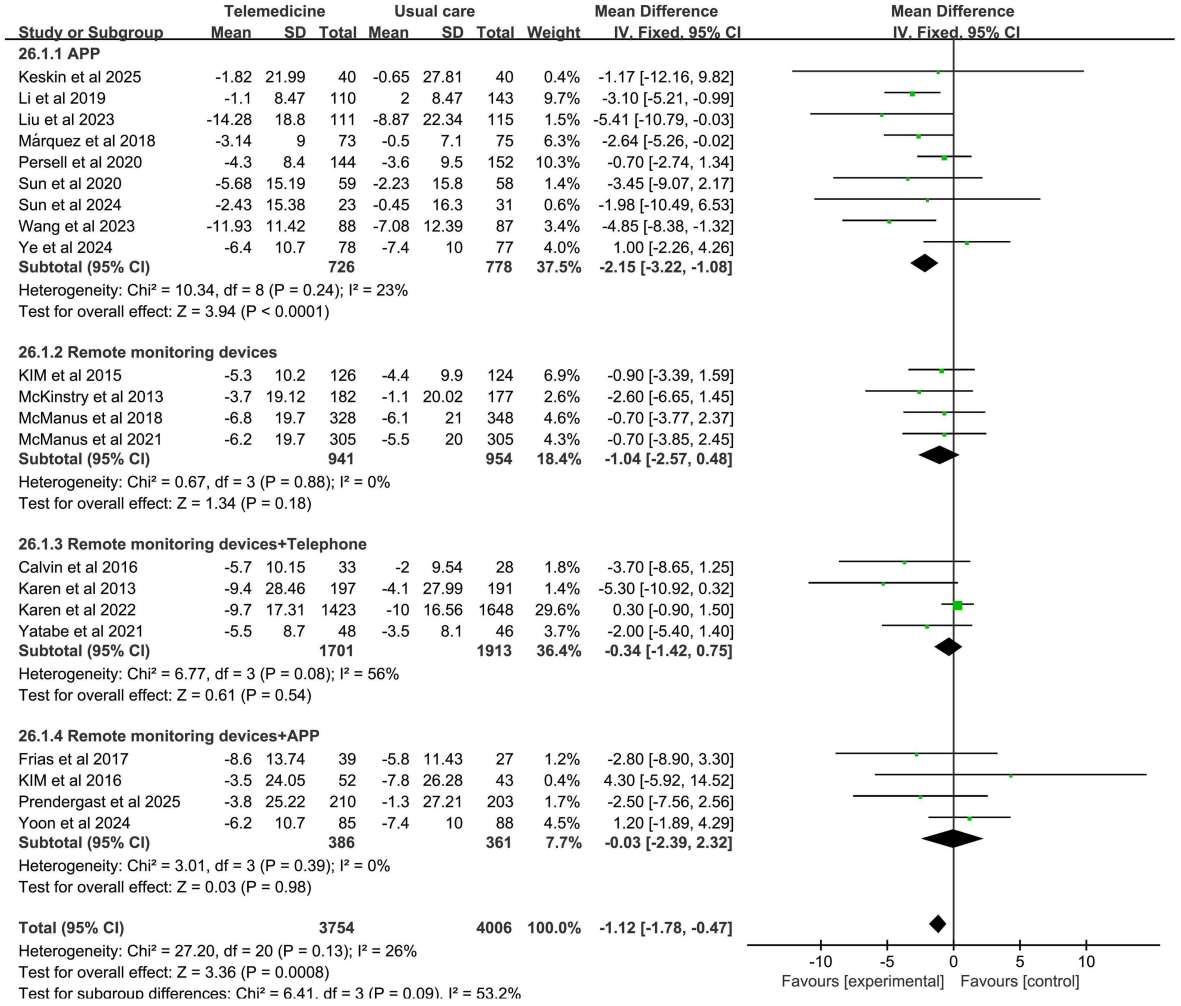
Subgroup analysis of intervention effects on DBP.

Subgroup analysis revealed that the APP group, remote monitoring devices group, remote monitoring devices + telephone group, and remote monitoring devices + APP group were all effective in reducing SBP, with statistically significant differences between groups (*z*-value 9.53, *p* < 0.001). Among these, the APP group (MD: −5.52; 95% CI: −6.85, −4.19; *p* = 0.03, *I*^2^ = 53%) and telemedicine + APP group (MD: −6.42; 95% CI: −9.28, −3.57; *p* = 0.46, *I*^2^ = 0%) demonstrated statistically significant reductions in SBP levels. However, the APP group exhibited high heterogeneity. The impact of the intervention method on DBP reduction demonstrated that the APP group effectively reduced DBP with no significant heterogeneity (MD: −2.15; 95% CI: −3.22, −1.08; *p* = 0.24, *I*^2^ = 23%). However, there was no statistically significant between-group difference. The results of the subgroup analysis on the effects of intervention modes on systolic and diastolic blood pressure are presented in Figures 5, 6.

#### 3.6.2 Duration

**Figure 7:**
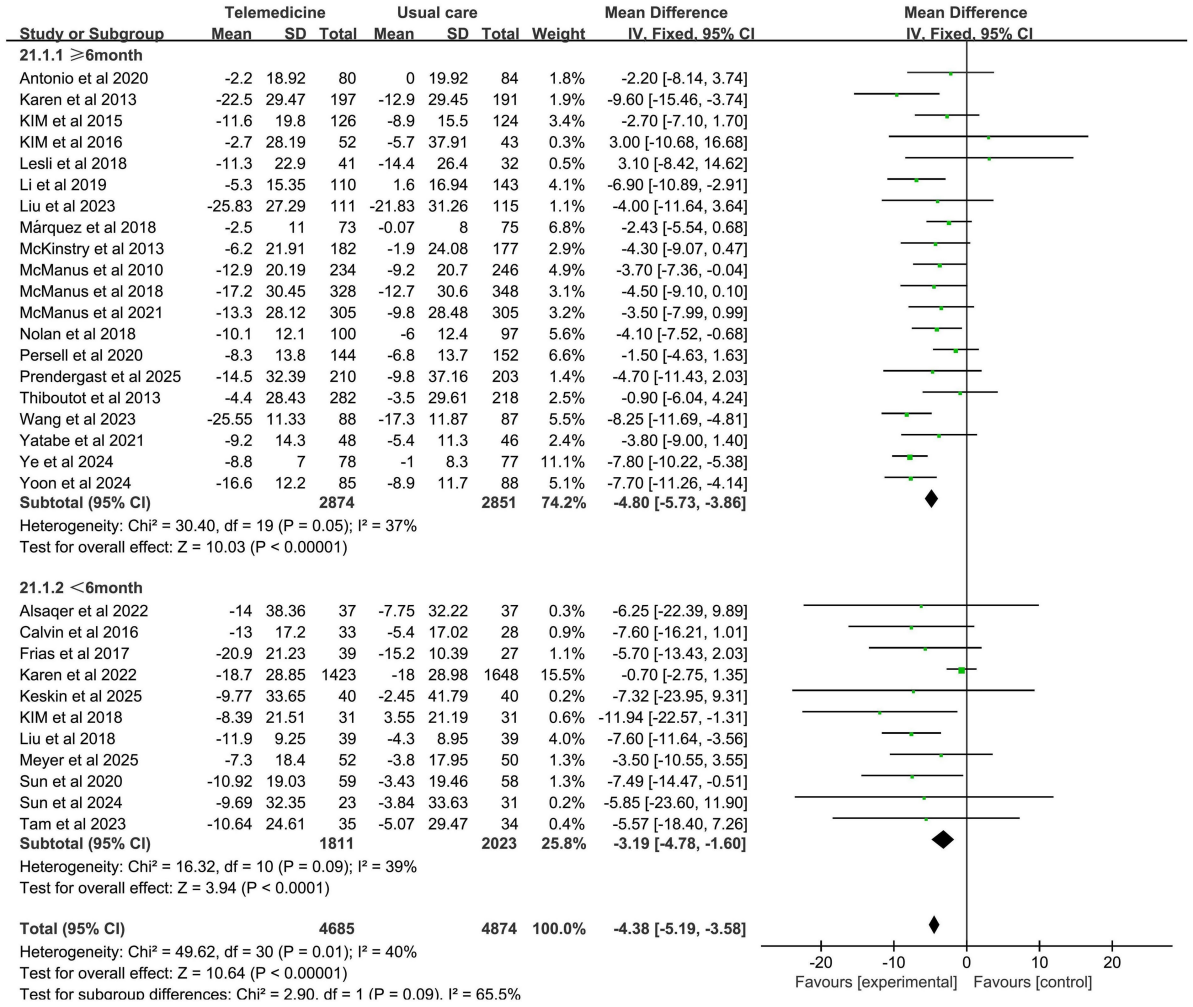
Subgroup analysis of duration effects on SBP.

**Figure 8:**
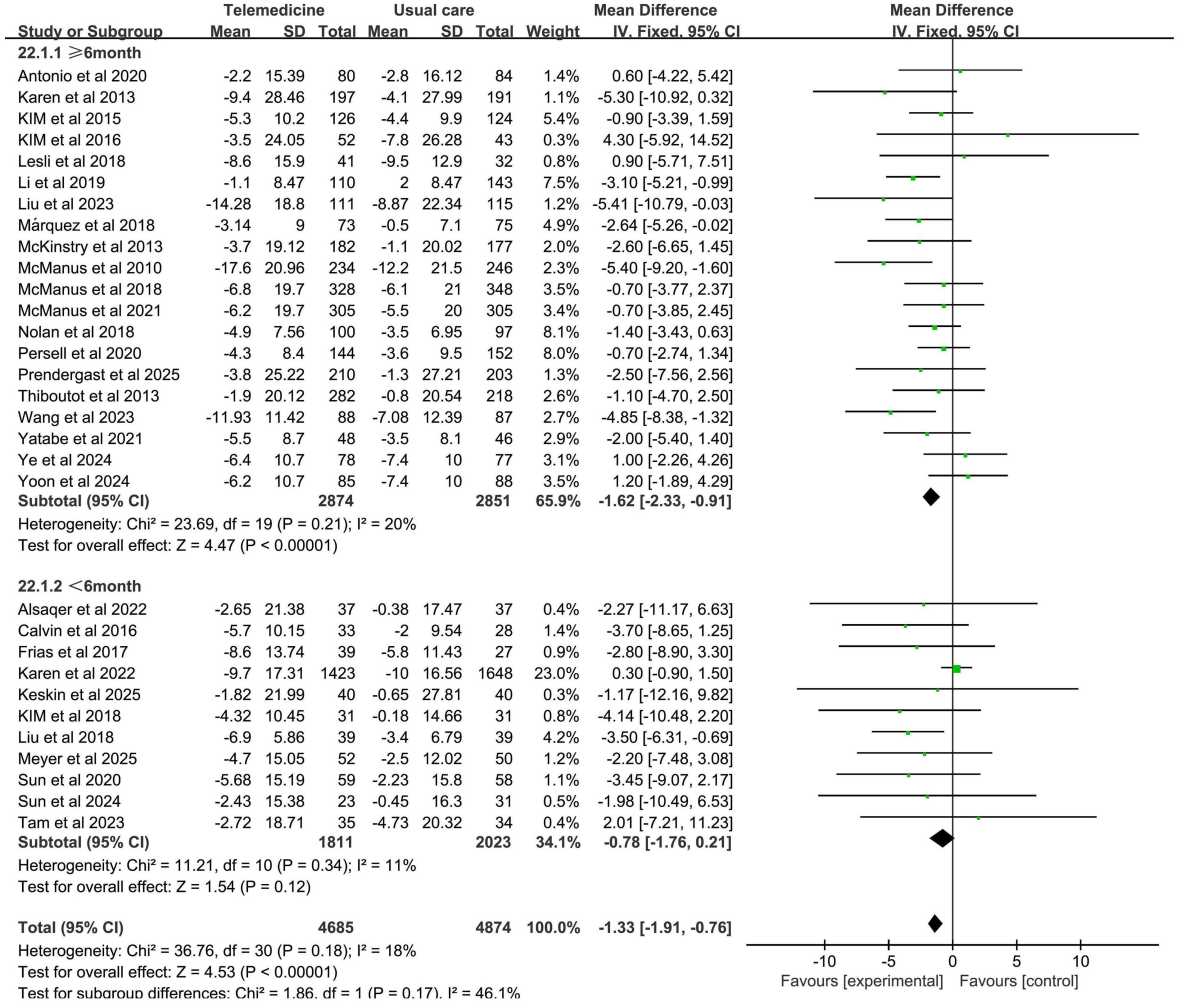
Subgroup analysis of duration effects on DBP.

Subgroup analysis revealed that the impact of intervention duration on SBP did not exhibit statistically significant disparities among the groups. The impact of intervention duration on DBP exhibited statistically significant disparities between groups (*z*-value 4.53, *p* < 0.001). In the cases where the intervention duration ≥6 months, a statistically significant reduction in DBP was observed, with no significant heterogeneity (MD: −1.62; 95% CI: −2.33, −0.91; *p* = 0.21, *I*^2^ = 20%). The results of the subgroup analysis on the effects of duration on systolic and diastolic blood pressure are presented in Figures 7, 8.

#### 3.6.3 The average age

**Figure 9:**
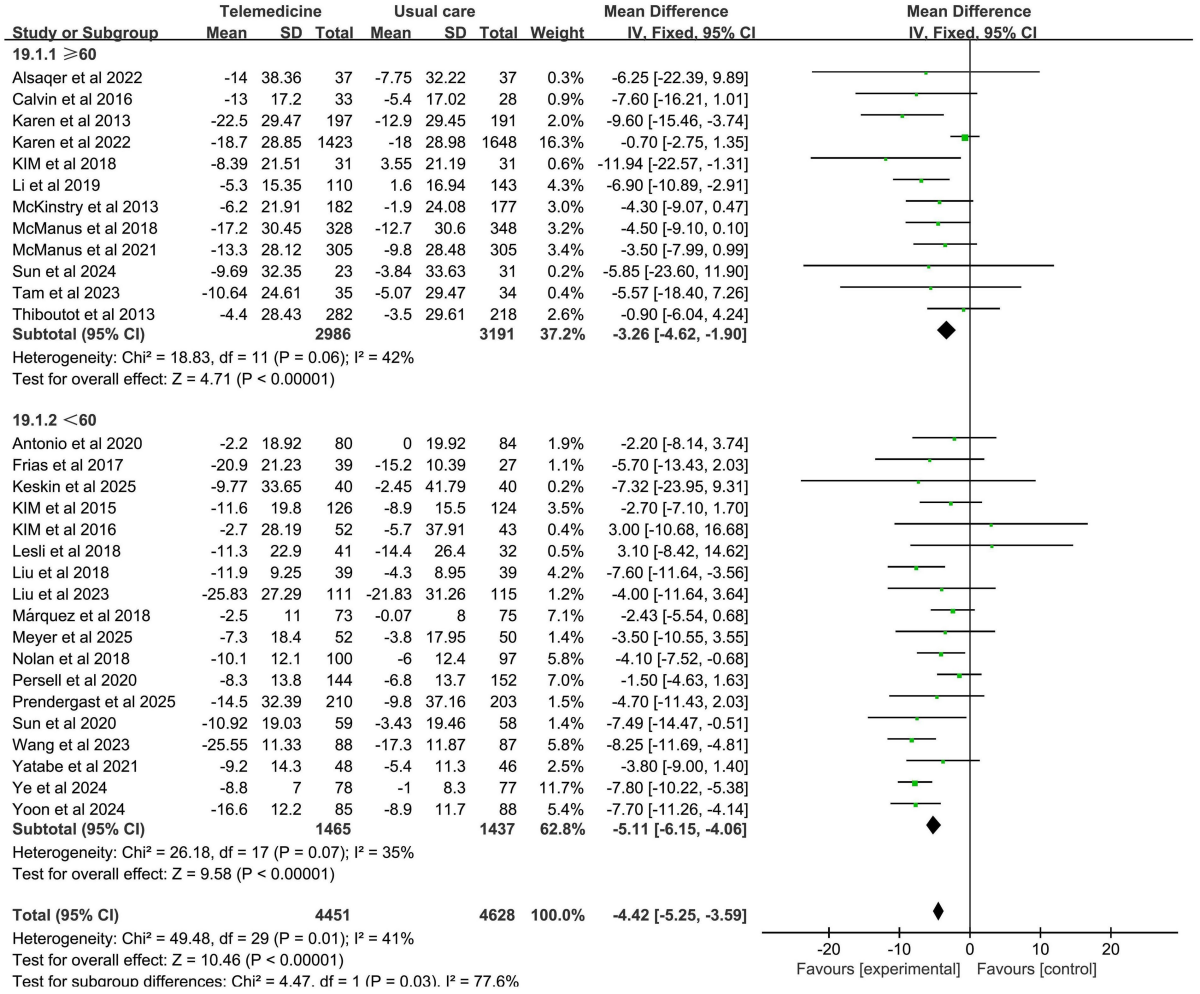
Subgroup analysis of the effects of mean age on SBP.

**Figure 10:**
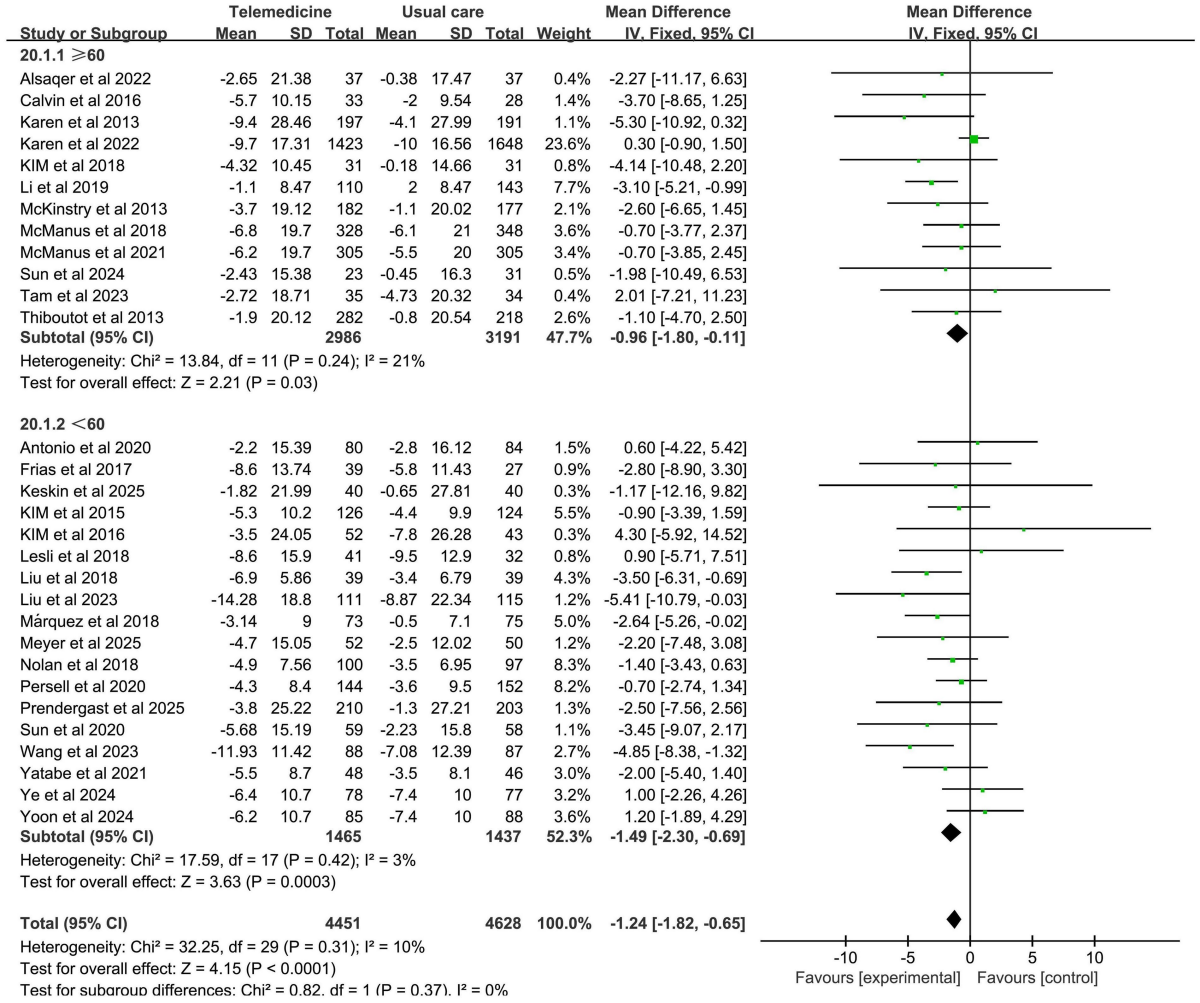
Subgroup analysis of the effects of mean age on DBP.

Subgroup analysis revealed a more pronounced reduction in SBP in the subgroup with an average age <60 years. However, there was mild heterogeneity (MD: −5.11; 95% CI: −6.15, −4.06; *p* = 0.07, *I*^2^ = 35%), with statistically significant differences between groups (*z*-value 10.46, *p* < 0.001). The investigation revealed no statistically significant differences in DBP between the groups with regard to the mean age. The results of the subgroup analysis on the effects of the average age on systolic and diastolic blood pressure are presented in Figures 9, 10.

#### 3.6.4 The region

In the subgroup analysis by region, all studies were divided into five parts according to region. The initial segment pertains to China, a nation that boasts distinctive characteristics with respect to its healthcare system, such as a hierarchical medical system and telemedicine policies, and hypertension epidemiology, such as high-salt diets and awareness rates. Consequently, a separate analysis is warranted from a clinical standpoint. The second region is North America, which includes studies from the United States and Canada. These two countries are geographically proximate, both high-income developed nations with mature telemedicine development, such as similar healthcare coverage and technology application models, resulting in high homogeneity within the group. The third section focuses on the European region, which includes the United Kingdom, Germany, and Spain. These three countries are all developed European nations with healthcare systems characterized by “universal healthcare coverage plus standardized chronic disease management.” The comparable telemedicine intervention approach is one that relies on general practitioner follow-ups. The fourth section is dedicated to the examination of other East Asian countries, including South Korea and Japan, which exhibit cultural and healthcare system similarities. The fifth group encompasses other regions, including Turkey, Jordan, and Peru. The three countries in question are located in the Middle East and South America, respectively, and they exhibit significant geographical and economic differences. However, the dearth of studies in this area has rendered it impracticable to form separate groups; thus, the studies have been combined for supplementary analysis.

**Figure 11:**
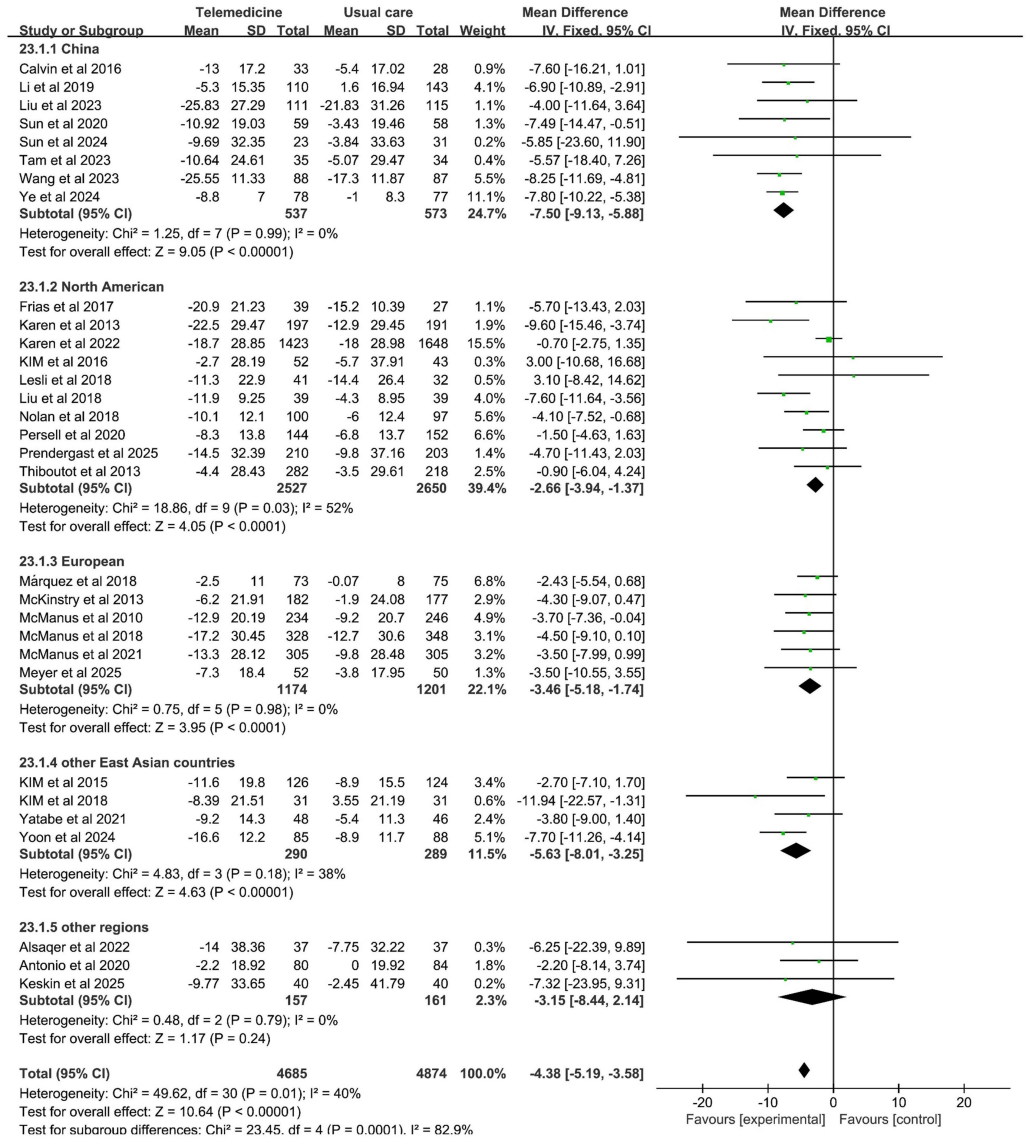
Subgroup analysis of the effects of the region on SBP.

**Figure 12:**
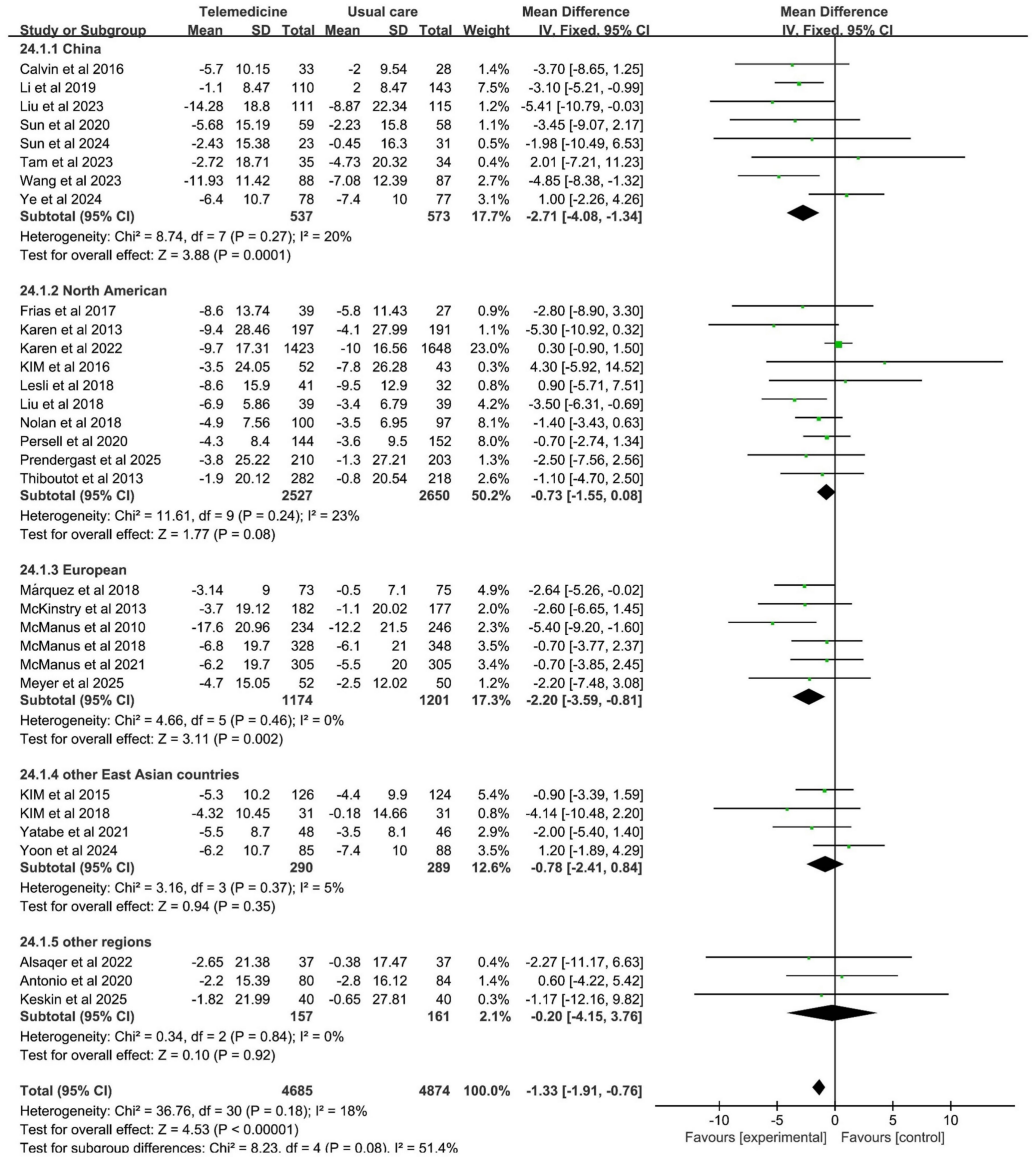
Subgroup analysis of the effects of the region on DBP.

Subgroup analysis revealed that the China, North American, European, and other East Asian subgroups all effectively reduced SBP, with statistically significant differences between subgroups (*z*-value 10.64, *p* < 0.001). Among these, the China subgroup (MD: −7.50; 95% CI: −9.13, −5.88; *p* = 0.99, *I*^2^ = 0%) and the other East Asian countries group (MD: −5.63; 95% CI: −8.01, −3.25; *p* = 0.18, *I*^2^ = 38%) demonstrated statistically significant reductions in SBP levels without heterogeneity. The investigation revealed no statistically significant disparities in DBP between the regions. However, the China group (MD: −2.71; 95% CI: −4.08, −1.34; *p* = 0.27, *I*^2^ = 20%) and the European group (MD: −2.20; 95% CI: −3.59, −0.81; *p* = 0.46, *I*^2^ = 0%) exhibited a more pronounced reduction in DBP with no significant heterogeneity. The results of the subgroup analysis on the effects of the region on systolic and diastolic blood pressure are presented in Figures 11, 12.

#### 3.6.5 Intervention content

This study categorizes telemedicine interventions into three main types for comparative analysis: education-focused (primarily delivering hypertension knowledge, medication guidance, and healthy lifestyle information through means such as article pushes or course offerings); monitoring and feedback-focused (emphasizing daily blood pressure monitoring and data upload by patients, with timely alerts or guidance during abnormal readings); and comprehensive support-focused (combining both education and monitoring while adding personalized behavioral guidance and long-term support). This classification facilitates exploration of the practical impact of different intervention content on blood pressure control.

**Figure 13:**
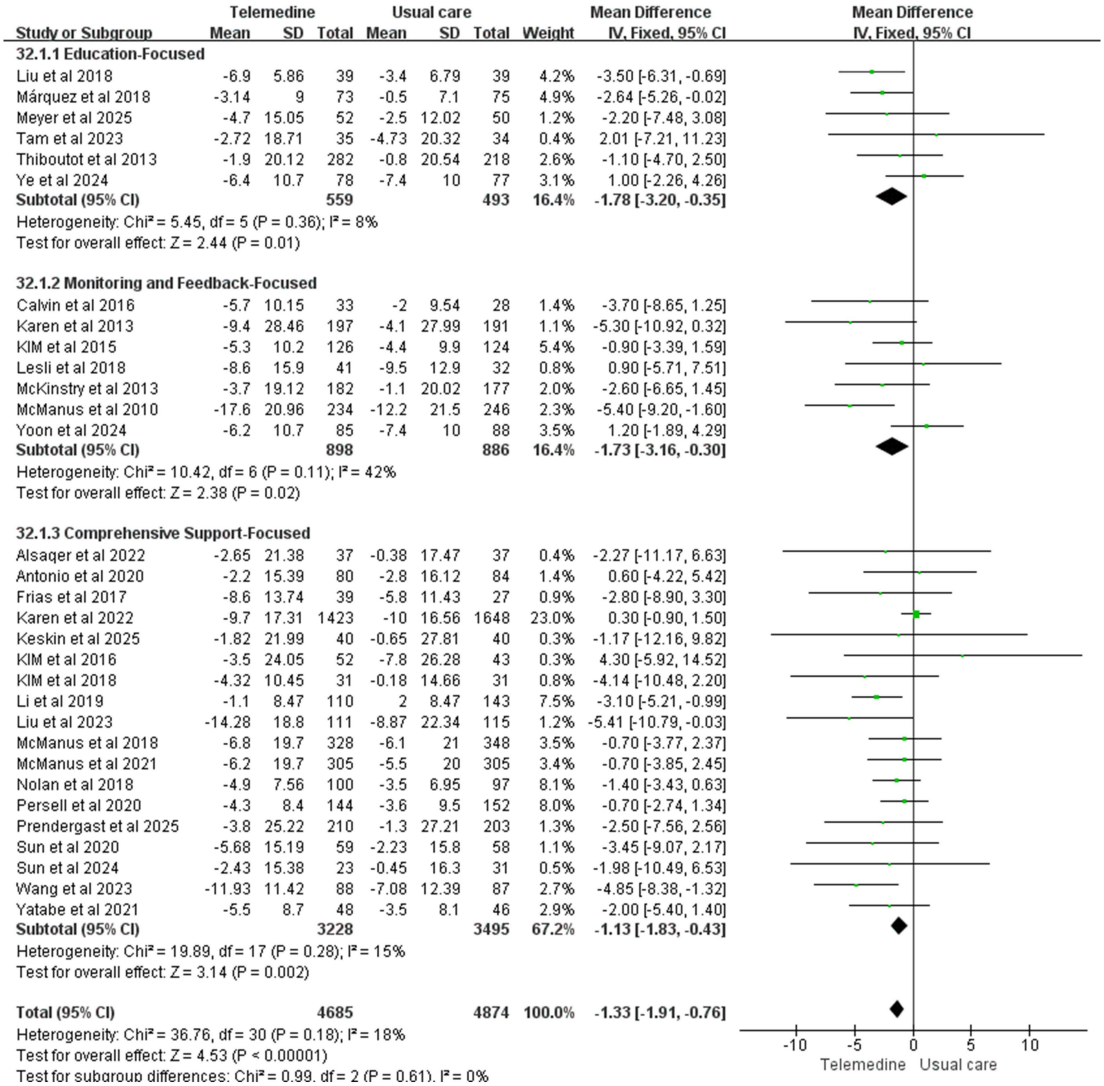
Subgroup analysis of intervention content on DBP.

**Figure 14:**
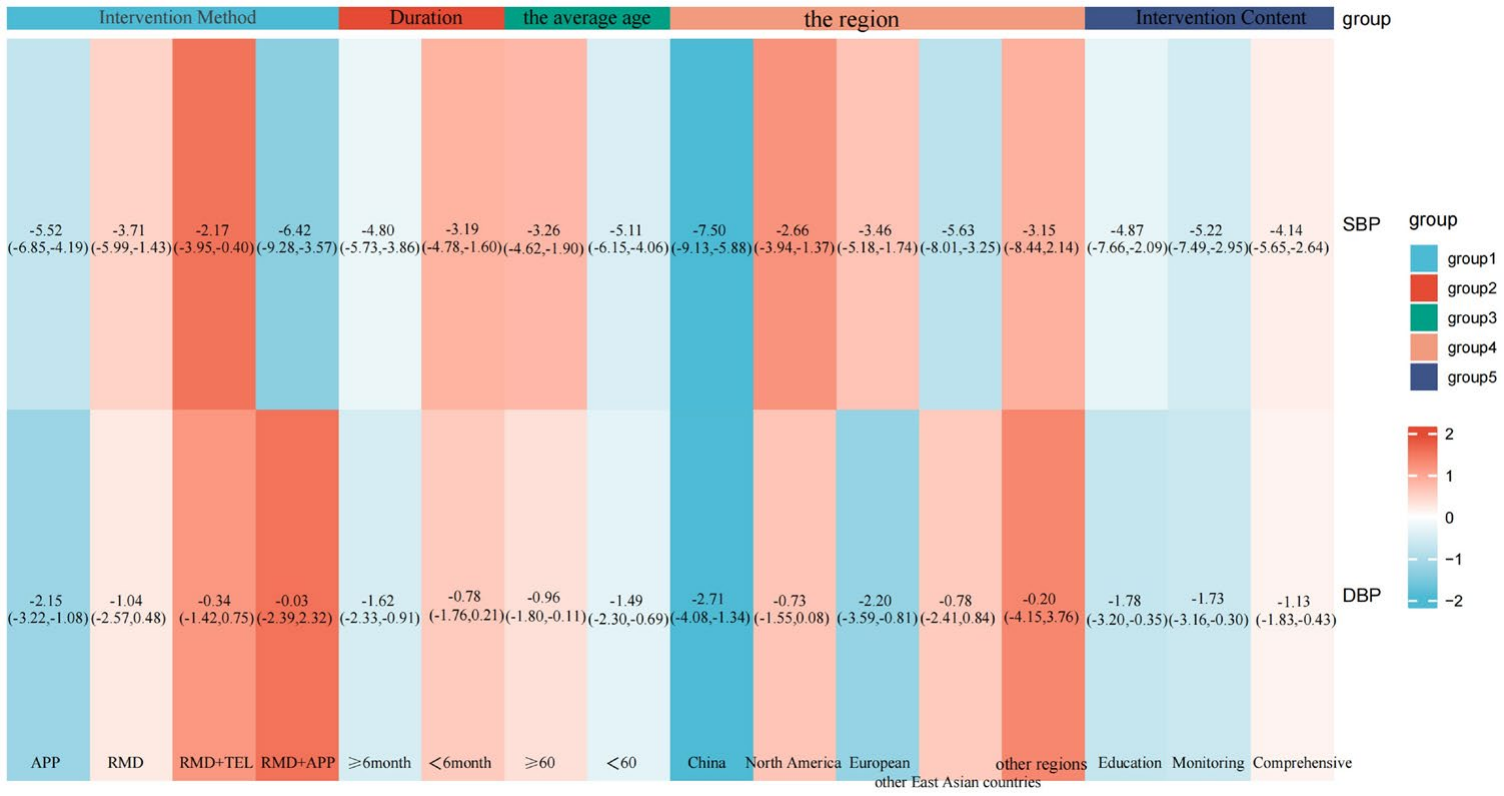
The heat plot of the subgroup analysis results for SBP and DBP.

**Figure 15:**
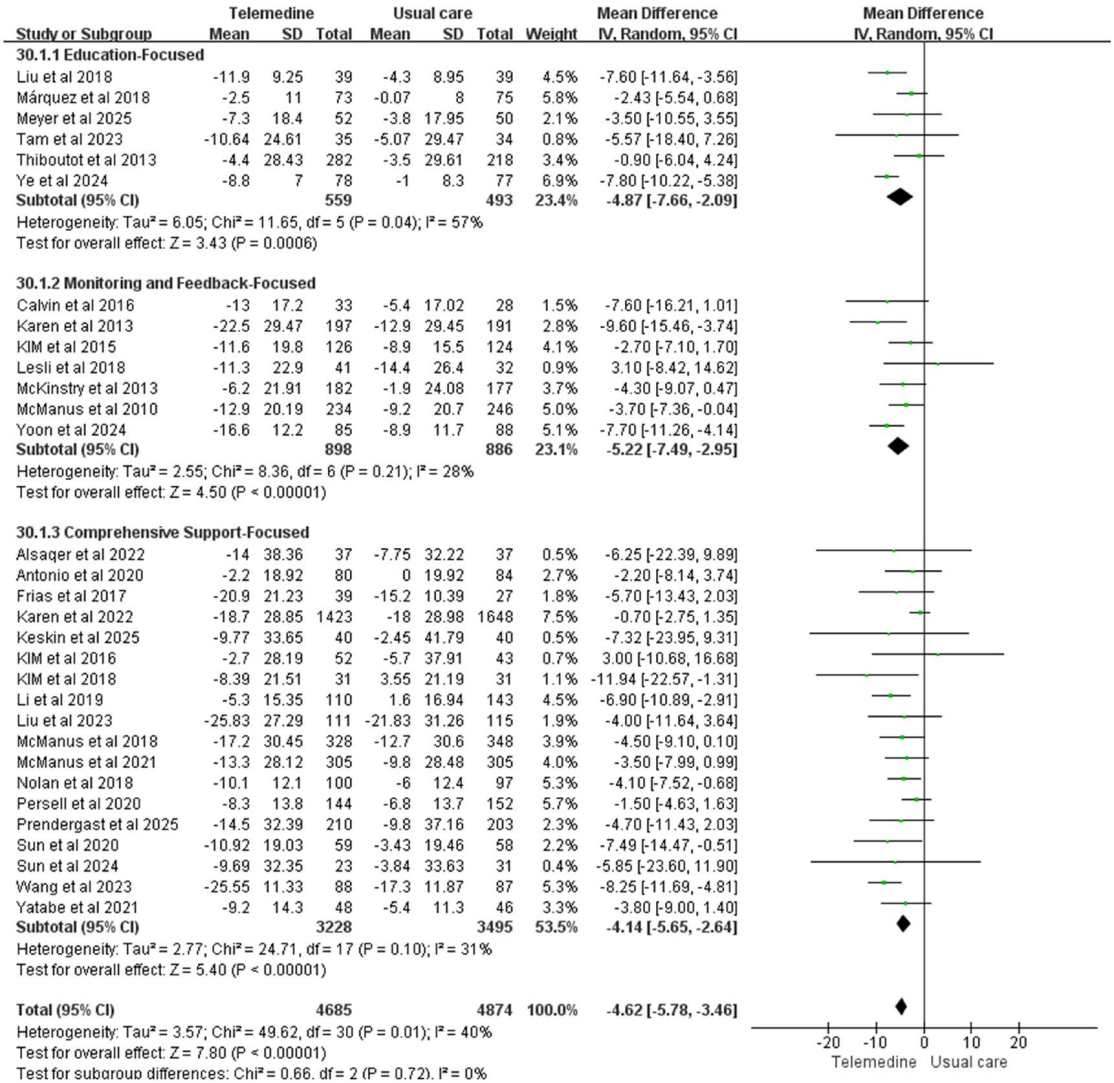
Subgroup analysis of intervention content on SBP.

Subgroup analysis revealed statistically significant reductions in both systolic and diastolic blood pressure across three subgroups: education-focused, monitoring and feedback-focused, and comprehensive support-focused. However, no statistically significant differences in blood pressure reduction were observed between subgroups with different intervention contents (SBP: MD: −4.62; 95% CI: −5.78, −3.46; *p* > 0.05, *I*^2^ = 0%; DBP: MD: −1.33; 95% CI: −1.91, −0.76; *p* > 0.05, *I*^2^ = 0%), suggesting comparable efficacy across different intervention content for blood pressure reduction. Figures 13, 14 presents the subgroup analysis results, illustrating the effects of different intervention content on systolic and diastolic blood pressure. The results of the subgroup analysis on the effects of intervention content on systolic and diastolic blood pressure are presented in Figures 13–15 displays the heat plot illustrating the results of the subgroup analysis for SBP and DBP. Funnel plots for the results of the subgroup analysis are provided in Supplementary Figure 3.

## 4 Discussion

This study evaluated the impact of telemedicine on blood pressure (primary outcome) in hypertensive patients while also examining its effects on medication adherence, hypertension knowledge score, self-efficacy, self-management and cardiovascular-metabolic composite indicators (e.g., weight, BMI, pulse pressure, TC and LDL). These secondary outcomes were included because they collectively form a multidimensional evaluation system for hypertension management effectiveness. Assessing telemedicine’s impact on these indicators facilitates a comprehensive understanding of its potential cardiovascular benefits.

The present study incorporated a total of 31 randomized controlled trials (RCTs), encompassing a collective sample of 9,559 adult patients diagnosed with hypertension. Meta-analysis results indicate that telemedicine interventions significantly reduce both systolic and diastolic blood pressure in patients. The magnitude of blood pressure reduction observed in this study (SBP decreased by 2.5–4.2 mmHg, DBP decreased by 1.5–2.4 mmHg) aligns with the efficacy of remote monitoring in hypertension management reported in previous studies (15–18, 26–28, 46, 47). Given the long-term consequences of hypertension, blood pressure reductions of this magnitude hold significant clinical value. Research indicates that in hypertensive populations, every 5 mmHg reduction in systolic blood pressure is associated with approximately a 10% decrease in the risk of major cardiovascular events (48). Furthermore, reductions in diastolic blood pressure are equally important. Studies confirm that even modest decreases (e.g., 2 mmHg) are associated with a significant reduction in the risk of fatal ischemic heart disease and stroke (45, 49). Therefore, if the blood pressure reduction observed in this study can be sustained long-term, it may translate into a significant reduction in cardiovascular disease risk. This finding may be closely related to the implementation of real-time monitoring and feedback mechanisms in telemedicine interventions, which have been demonstrated to enhance patient compliance and self-management capabilities, thereby effectively controlling blood pressure levels (50, 51).

Compared to previous systematic reviews that primarily focused on blood pressure outcomes, this study’s analysis further reveals that telemedicine also offers significant benefits in enhancing patients’ knowledge of hypertension, self-efficacy, and self-management capabilities. Results based on hypertension knowledge suggest that telemedicine interventions can effectively enhance patients’ disease knowledge and promote their health management capabilities. The enhancement of patients’ comprehension regarding hypertension and its management strategies has been demonstrated to engender heightened motivation to engage in treatment regimens, thereby leading to the optimization of treatment outcomes. This finding aligns with the findings of previous research, which indicates that health education is an important component of improving chronic disease management (52, 53). According to social cognitive theory, self-efficacy is a core driver of health behavior change (54) and is positively correlated with health behaviors (55). Furthermore, self-efficacy theory finds extensive application in the fields of mental health and chronic disease management (56). Furthermore, enhanced patient engagement and self-management capabilities are closely associated with improved clinical outcomes, promoting better adherence and blood pressure control (57). Indicating that these interventions are effective not only at lowering blood pressure but also at improving patients’ general health and quality of life (8, 58–60). The significant improvement in these patient empowerment indicators represents not only a direct benefit of telemedicine but also likely serves as a key mediating mechanism for achieving effective blood pressure control. This finding systematically reveals the added value of telemedicine beyond mere blood pressure monitoring, marking a crucial advancement compared to earlier reviews that focused solely on physiological indicators.

Regarding metabolic indicators, this study observed that telemedicine significantly reduced pulse pressure. Elevated pulse pressure has been confirmed by multiple epidemiological studies, including the Framingham Cohort, as a strong independent predictor of coronary heart disease and heart failure risk (61, 62). Therefore, this reduction holds clear clinical significance. Moreover, the intervention group exhibited a statistically significant decrease in cholesterol levels compared to the control group. This finding is consistent with existing literature, which suggests that in hypertension management, the primary focus should be on monitoring and intervening in lipid metabolism to reduce the risk of cardiovascular events (63, 64). We speculate that the improvement in lipid profiles (TC/LDL) through telemedicine may primarily be an indirect effect, achieved by promoting lifestyle changes such as healthy eating and weight reduction. Seeking effective weight management strategies through telemedicine remains a reasonable approach, as it has been proven to reduce cardiovascular risk (65). However, variations in dietary education content, intervention depth, and intensity across different studies have led to significant variability in lipid-lowering effects, with their overall clinical significance remaining unclear. The implementation of systematic health management strategies, combined with the integration of telemedicine technologies, holds promise for providing patients with more comprehensive treatment options and improving their long-term health outcomes.

Although no statistically significant difference in LDL reduction was observed in this study, but the magnitude of reduction observed in the main analysis is of interest, as large-scale meta-analyses have established that a sustained reduction of ~1 mmol/L in LDL-cholesterol is associated with a substantial ~20% reduction in major vascular events (66). This suggests that telemedicine, if optimized to consistently deliver such effects, could contribute to long-term cardiovascular risk mitigation.

Regarding medication adherence, this study found no statistically significant improvement attributable to telemedicine, with high heterogeneity observed. This discrepancy may be attributed to the diversity of telemedicine intervention methods and the lack of uniformity in medication adherence measurement tools. While statistically non-significant, the direction of effect is encouraging given the established link between medication adherence and improved cardiovascular outcomes (67, 68). Notably, Mulè et al. (46) emphasize that self-monitoring of blood pressure can significantly improve patients’ adherence to lifestyle changes and medication by encouraging their active participation in their own care (69). Therefore, subsequent studies should delve deeper into optimizing medication management within telemedicine interventions to enhance patients’ overall treatment outcomes.

Subgroup analysis revealed that the intervention method combining remote monitoring devices with applications led to a more substantial reduction in SBP in younger patients (mean age <60 years). This outcome may be attributable to the heightened acceptance and inclination towards technology exhibited by younger patients. Recent studies have demonstrated that younger individuals exhibit a greater propensity to employ contemporary technologies in their health management practices. This utilization of technology facilitates rapid access to pertinent health information, which in turn fosters the adoption of proactive health behaviors. This finding underscores the importance of individual differences in intervention outcomes, suggesting that when developing intervention strategies for patients of different age groups, we should consider variations in their technological acceptance and usage habits. The advent of advanced 5G, the Internet of Things, and big data technologies has precipitated the emergence of novel and efficacious methodologies for the management of chronic diseases. However, it is imperative to acknowledge the heterogeneity in the acceptance of new technologies across different demographic groups, particularly among the older adults and those with limited educational attainment. The adoption of new technologies may present significant challenges for these groups. The issue of health equity persists as a pivotal consideration. On a global scale, chronic diseases such as hypertension are predominantly prevalent among middle-aged and older adults. The effective management of these conditions in this population remains a significant challenge.

During the intervention, it is also imperative to provide personalized recommendations for blood pressure management based on patient feedback (47). The intervention methods of the telemedicine joint application have been demonstrated to enhance the convenience of adjusting antihypertensive medications and implementing personalized lifestyle interventions, thereby achieving enhanced effectiveness in reducing blood pressure. This observation is in alignment with the findings reported in other studies (70, 71). Despite the findings of research indicating that augmenting the duration of the intervention does not result in a substantial reduction of blood pressure in hypertensive patients. However, the research report indicates that patients can adhere to proper treatment regimens under prolonged intervention. Consequently, the implementation of health intervention measures should be initiated at the earliest possible stage and sustained over an extended period to cultivate effective blood pressure management practices. This approach is aimed at enhancing the quality of life for patients afflicted with long-term hypertension. Subgroup analyses of intervention content revealed that telemedicine interventions with different content (such as education-focused, monitoring and feedback-focused or comprehensive support-focused), all effectively reduced blood pressure, though no statistically significant differences in outcomes were observed between groups. This suggests potential “equivalence” among different telemedicine intervention content. This may stem from the shared foundation of sustained support, supervision, and physician-patient interaction provided by telemedicine, which enables the efficacy of various interventions. Therefore, clinical practice should prioritize the “quality of implementation” over the “number of interventions.” Decision-makers can flexibly select or design the most appropriate intervention strategy based on practical factors such as cost, accessibility, and patient preferences.

In adult patients with hypertension, telemedicine has shown considerable effectiveness in lowering SBP and DBP, according to preliminary research. Additionally, it has been discovered that these interventions improve patients’ self-efficacy and self-management abilities. In order to promote health equity, health policies can be developed and implemented in a way that is specific to these interventions. Vulnerable populations who face obstacles to traditional care may benefit greatly from increased access to digital health resources. These demographics include low-income people, members of marginalized groups, and people living in rural areas. In order to effectively address health inequities, policy frameworks should place a high priority on incorporating remote health into standard hypertension treatment procedures. To encourage fair uptake and continued engagement across a range of demographics, such tactics could involve making sure there is sufficient broadband infrastructure, supplying the required equipment, providing multilingual and culturally appropriate support, and setting up long-term compensation systems. The potential of telemedicine treatments can be optimized by taking these factors into account. This will improve clinical outcomes, lessen disparities, and achieve long-term health equity in the treatment of hypertension.

## 5 Limitations

It is crucial to remember that this study has limitations. Although all of the included studies were RCTs, there were several methodological issues because of the nature of the intervention techniques, including unclear information on allocation concealment and the absence of outcome assessor blinding. Additionally, the duration and manner of the therapies varied from study to study, which resulted in variations in the meta-analysis. The survey results must be interpreted and analyzed with these restrictions in mind. Notwithstanding these drawbacks, the unique study design and data-gathering techniques have, in part, increased the results’ dependability and provided valuable benchmarks for further research. By expanding the sample size, prolonging the intervention period, and using a more exacting randomized design, future research may be able to get beyond these restrictions. These adjustments would help to confirm and broaden the study’s findings’ relevance.

## 6 Conclusion

The results of this study indicate that there is a great deal of clinical potential for using telemedicine to manage hypertension. SBP and DBP have been demonstrated to significantly decrease when telemedicine interventions are used for persons with hypertension who are 18 years of age and older. The average drop in blood pressure was 4.62 mmHg for SBP and 1.33 mmHg for DBP, which was deemed to be noteworthy. These results, which show the significant clinical value of telemedicine interventions in the management of hypertension, are based on a thorough examination of several RCTs. Additionally, improvements in self-efficacy and self-management have been noted. In order to improve patient adherence and health outcomes, doctors should actively investigate integrating telemedicine interventions into hypertension care, according to this finding, which offers substantial support for clinical practice.

Although the benefits of telemedicine interventions have been shown in this study, there are a number of topics that should be investigated further in subsequent studies. First, it is advised that the efficacy of telemedicine interventions be further validated across various populations, especially by means of subgroup analyses that focus on patients with diverse comorbidities, ages, and genders. Second, to improve customized management techniques for hypertension, future study might examine the best mix of various telemedicine intervention formats and their effects on patients’ capacity for self-management. By filling in knowledge gaps in the body of existing literature, this research will increase our understanding of the management of hypertension.

## Data Availability

The original contributions presented in the study are included in the article/Supplementary material, further inquiries can be directed to the corresponding author.
