# Supplementary material for: Effectiveness of telemedicine interventions on blood pressure control and self-management efficacy in hypertensive patients: a systematic review and meta-analysis
Source: Front Public Health. 2026 Jan 12;13:1693141. doi: 10.3389/fpubh.2025.1693141 (PMC12833360; doi:10.3389/fpubh.2025.1693141)
Supplement: Supplementary file 1 [file Data_Sheet_1.docx]

Supplementary Material

**1 Supplementary Tables**

**Supplementary Table 1. Specific search strategies for each database**

**Supplementary Table 2. Excluded full-text with reason**

**2 Supplementary Figures**

**Supplementary Figure1. Forest Plots for the other Various Outcome Measures.**

**Supplementary Figure2. Funnel Plots for Assessment of Publication Bias Across All Outcome Measures**

**Supplementary Figure3. Funnel Plots Assessing Publication Bias for Subgroup Analyses of Systolic and Diastolic Blood Pressure**

**Supplementary Table 1. Specific search strategies for each database**

| Database | Search Terms | Result |
| --- | --- | --- |
| **Pubmed (Date: May 24, 2025)** | | |
| No. |  | |
| #1 | (((("Hypertension"[Mesh]) OR (Blood Pressure, High[Title/Abstract])) OR (Blood Pressures, High[Title/Abstract])) OR (High Blood Pressure[Title/Abstract])) OR (High Blood Pressures[Title/Abstract]) | 341,660 |
| #2 | (((((("Self-Management"[Mesh]) OR (Self Management)) OR (Management, Self)) OR (Self-Management Programs)) OR (Program, Self-Management)) OR (Self-Management Program)) OR (Self Management Programs) | 130,401 |
| #3 | ((((((((((((((((("Telemedicine"[Mesh]) OR (Virtual Medicine)) OR (Medicine, Virtual)) OR (Tele-Referral)) OR (Tele Referral)) OR (Tele-Referrals)) OR (Mobile Health)) OR (Health, Mobile)) OR (mHealth)) OR (Telehealth)) OR (eHealth)) OR (Tele-Intensive Care)) OR (Tele Intensive Care)) OR (Tele-ICU)) OR (Tele ICU)) OR (Telecare)) OR (Tele-Care)) OR (Tele Care) | 174,166 |
| #4 | #1 AND #2 AND #3 | 312 |
| **Web of Science (Date: May 24, 2025)** | | |
| No. |  | |
| #1 | ((((TS=(Hypertension)) OR TS=(Blood Pressure, High)) OR TS=(Blood Pressures, High)) OR TS=(High Blood Pressure)) OR TS=(High Blood Pressures) | 1,170,004 |
| #2 | ((((((TS=(Self-Management)) OR TS=(Self Management)) OR TS=(Management, Self)) OR TS=(Self-Management Programs)) OR TS=(Program, Self-Management)) OR TS=(Self-Management Program)) OR TS=(Self Management Programs) | 299,122 |
| #3 | (((((((((((((((((TS=(Telemedicine)) OR TS=(Virtual Medicine)) OR TS=(Medicine, Virtual)) OR TS=(Tele-Referral)) OR TS=(Tele Referral)) OR TS=(Tele-Referrals)) OR TS=(Mobile Health)) OR TS=(Health, Mobile)) OR TS=(mHealth)) OR TS=(Telehealth)) OR TS=(eHealth)) OR TS=(Tele-Intensive Care)) OR TS=(Tele Intensive Care)) OR TS=(Tele-ICU)) OR TS=(Tele ICU)) OR TS=(Telecare)) OR TS=(Tele-Care)) OR TS=(Tele Care) | 202,085 |
| #4 | #1 AND #2 AND #3 | 1,053 |
| **Embase (Date: May 24, 2025)** | | |
| No. |  | |
| #1 | 'hypertension'/exp | 1,157,240 |
| #2 | 'acute hypertension':ti,ab,kw OR 'arterial hypertension':ti,ab,kw OR 'blood pressure, high':ti,ab,kw OR 'cardiovascular hypertension':ti,ab,kw OR 'controlled hypertension':ti,ab,kw OR 'endocrine hypertension':ti,ab,kw OR 'high blood pressure':ti,ab,kw OR 'high renin hypertension':ti,ab,kw OR 'htn (hypertension)':ti,ab,kw OR 'hypertensive disease':ti,ab,kw OR 'hypertensive effect':ti,ab,kw OR 'hypertensive reaction':ti,ab,kw OR 'hypertensive response':ti,ab,kw OR 'neurogenic hypertension':ti,ab,kw OR 'preexistent hypertension':ti,ab,kw OR 'salt high blood pressure':ti,ab,kw OR 'salt hypertension':ti,ab,kw OR 'secondary hypertension':ti,ab,kw OR 'systemic hypertension':ti,ab,kw OR 'hypertension':ti,ab,kw | 852,990 |
| #3 | #1 OR #2 | 1,406,309 |
| #4 | 'self care'/exp | 119,196 |
| #5 | 'self management':ti,ab,kw OR 'self treatment':ti,ab,kw OR 'self-management':ti,ab,kw OR 'self-nurturance':ti,ab,kw OR 'selfcare':ti,ab,kw OR 'selfmanagement':ti,ab,kw OR 'selftreatment':ti,ab,kw OR 'self care':ti,ab,kw | 86,742 |
| #6 | #4 OR #5 | 143,243 |
| #7 | 'telemedicine'/exp | 86,339 |
| #8 | 'tele medicine':ti,ab,kw OR 'virtual medicine':ti,ab,kw OR 'telemedicine':ti,ab,kw | 40,484 |
| #9 | #7 OR #8 | 94,034 |
| #10 | #3 AND #6 AND #9 | 357 |
| **Cochrane Library (Date: May 24, 2025)** | | |
| No. |  | |
| #1 | MeSH descriptor: [Hypertension] explode all trees | 25,238 |
| #2 | (Blood Pressures, High):ti,ab,kw OR (High Blood Pressure):ti,ab,kw OR (High Blood Pressures):ti,ab,kw OR (Blood Pressure, High):ti,ab,kw | 31,636 |
| #3 | #1 OR #2 | 52,691 |
| #4 | MeSH descriptor: [Self-Management] explode all trees | 1,378 |
| #5 | (Program, Self-Management or Self-Management Programs or Self Management Programs or Self-Management Program or Self Management or Management, Self or Self Management):ti,ab,kw (Word variations have been searched) | 34,298 |
| #6 | #4 OR #5 | 34,298 |
| #7 | MeSH descriptor: [Telemedicine] explode all trees | 5,312 |
| #8 | (Medicine, Virtual or Tele-Referrals or Tele Referral or Virtual Medicine or Tele-Referral or Tele-Intensive Care or Tele ICU or Tele Intensive Care or Tele-ICU or Telecare or Tele-Care or Tele Care or Health, Mobile or mHealth or Mobile Health or Telehealth or eHealth):ti,ab,kw (Word variations have been searched) | 26,720 |
| #9 | #7 OR #8 | 29,376 |
| #10 | #3 AND #6 AND #9 | 322 |

**Supplementary Table 2. Excluded full-text with reasons**

| No. | References | Reason for exclusion |
| --- | --- | --- |
| 1 | Greene LK 2024^1^ | Not RCTs |
| 2 | Xu L 2017^2^ | Study protocol |
| 3 | Migneault JP 2012^3^ | Incomplete data |
| 4 | Polgreen LA 2020^4^ | Study protocol |
| 5 | Golbus JR 2024^5^ | Study protocol |
| 6 | Kao CW 2019^6^ | Incomplete data |
| 7 | Lewinski AA 2019^7^ | Not RCTs |
| 8 | Kerby TJ 2012^8^ | Wrong outcomes |
| 9 | Beger C 2023^9^ | Not RCTs |
| 10 | Ware P 2022^10^ | Wrong population |
| 11 | Margolis KL 2012^11^ | Study protocol |
| 12 | Gupta A 2024^12^ | Study protocol |
| 13 | Zhang H 2024^13^ | Study protocol |
| 14 | Irizarry T 2018^14^ | Not RCTs |
| 15 | Maciejewski ML 2014^15^ | Incomplete data |
| 16 | Dehmer SP 2018^16^ | Incomplete data |
| 17 | Castela Forte J 2022^17^ | Not RCTs |
| 18 | Oh SW 2022^18^ | Wrong population |
| 19 | Wakefield BJ 2014^19^ | Incomplete data |
| 20 | Thakar V 2023^20^ | Study protocol |
| 21 | Lee MC 2024^21^ | Incomplete data |
| 22 | Vestala H 2024^22^ | Repeated publications |
| 23 | Liu S 2020^23^ | Incomplete data |
| 24 | Avegno KS 2023^24^ | Not RCTs |
| 25 | Davidson TM 2015^25^ | Incomplete data |
| 26 | Lokker C 2021^26^ | Study protocol |
| 27 | Meurer WJ 2019^27^ | Incomplete data |
| 28 | White WB 2011^28^ | Incomplete data |
| 29 | Bosworth HB 2007^29^ | Incomplete data |
| 30 | Piette JD 2012^30^ | Incomplete data |
| 31 | Chandler J 2019^31^ | Incomplete data |
| 32 | Jiang Y 2024^32^ | Incomplete data |
| 33 | Gupta A 2023^33^ | Incomplete data |
| 34 | Bengtsson U 2016^34^ | Not RCTs |
| 35 | Buis LR 2019^35^ | Study protocol |
| 36 | Oh SH 2024^36^ | Wrong outcomes |
| 37 | Lakshminarayan K 2021^37^ | Study protocol |
| 38 | Varleta P 2017^38^ | Incomplete data |
| 39 | Jackson GL 2016^39^ | Study protocol |
| 40 | Wu D 2021^40^ | Not RCTs |
| 41 | Midlöv P 2020^41^ | Study protocol |
| 42 | Andersson U 2023^42^ | Incomplete data |
| 43 | Jackson GL 2012^43^ | Incomplete data |
| 44 | Bosworth HB 2011^44^ | Incomplete data |
| 45 | Meurer WJ 2020^45^ | Study protocol |
| 46 | Hermansson-Borrebaeck R 2025^46^ | Repeated publications |
| 47 | Bobrie G 2007^47^ | Not RCTs |
| 48 | Li Y 2022^48^ | Study protocol |
| 49 | Prendergast HM 2021^49^ | Study protocol |
| 50 | Buis L 2017^50^ | Incomplete data |
| 51 | Jung H 2017^51^ | Not RCTs |
| 52 | Yatabe MS 2018^52^ | Study protocol |
| 53 | Lee HY 2019^53^ | Incomplete data |
| 54 | Wong AKC 2023^54^ | Wrong outcomes |
| 55 | Moon EW 2019^55^ | Wrong outcomes |
| 56 | Bosworth HB 2009^56^ | Incomplete data |
| 57 | Grant S 2019^57^ | Incomplete data |
| 58 | Fitzpatrick AL 2019^58^ | Study protocol |
| 59 | Bilger M 2021^59^ | Incomplete data |
| 60 | Zhang X 2020^60^ | Wrong outcomes |
| 61 | Sawitree Visanuyothin 2017^61^ | Dissertation |
| 62 | Rubinstein A 2016^62^ | Wrong population |
| 63 | Pletcher MJ 2022^63^ | Non-standard control group |
| 64 | Rowland SA 2022^64^ | Wrong outcomes |
| 65 | Zare S 2019^65^ | Not RCTs |
| 66 | Lokker C 2021^66^ | Study protocol |
| 67 | Persell SD 2018^67^ | Study protocol |
| 68 | Parati G 2009^68^ | Incomplete data |
| 69 | Eldawati E 2022^69^ | Not RCTs |
| 70 | Davidson TM 2015^70^ | Wrong outcomes |
| 71 | Bilger M 2021^71^ | Incomplete data |
| 72 | Hermansson-Borrebaeck R 2023^72^ | Wrong outcomes |
| 73 | McManus RJ 2009^73^ | Study protocol |
| 74 | Bosworth HB 2007^74^ | Study protocol |
| 75 | Aigbonoga D 2025^75^ | Wrong outcomes |
| 76 | Ashoorkhani M 2016^76^ | Study protocol |
| 77 | Band R 2016^77^ | Study protocol |
| 78 | Bernocchi P 2014^78^ | Not RCTs |
| 79 | Bozorgi A 2021^79^ | Wrong outcomes |
| 80 | Debon R 2020^80^ | Not RCTs |
| 81 | Erden Y 2025^81^ | Wrong outcomes |
| 82 | Franssen M 2017^82^ | Study protocol |
| 83 | Gazit T 2021^83^ | Not RCTs |
| 84 | Hammersley V 2020^84^ | Not RCTs |
| 85 | Hartch CE 2024^85^ | Wrong outcomes |
| 86 | Ionov MV 2020^86^ | Not English |
| 87 | Kwan YH 2024^87^ | Study protocol |
| 88 | Li T 2019^88^ | Study protocol |
| 89 | Margolis KL 2022^89^ | Wrong outcomes |
| 90 | McManus RJ 2009^90^ | Study protocol |
| 91 | Parati G 2013^91^ | Study protocol |
| 92 | Pati S 2025^92^ | Study protocol |
| 93 | Schlichtiger J 2024^93^ | Study protocol |
| 94 | Shen X 2021^94^ | Study protocol |
| 95 | Taher M 2021^95^ | Study protocol |
| 96 | Wang Z 2021^96^ | Study protocol |

**References**

1 Greene LK, Song G, Palma AV, et al. A Case Series Study Assessing an Equity-Focused Implementation of Self-Monitoring Blood Pressure Programs Using Telehealth. *J Public Health Manag Pract*. 2024;30:S71-S79.

2 Xu L, Fang WY, Zhu F, et al. A coordinated PCP-Cardiologist Telemedicine Model (PCTM) in China's community hypertension care: study protocol for a randomized controlled trial. *Trials*. 2017;18(1):236.

3 Migneault JP, Dedier JJ, Wright JA, et al. A culturally adapted telecommunication system to improve physical activity, diet quality, and medication adherence among hypertensive African-Americans: a randomized controlled trial. *Ann Behav Med*. 2012;43(1):62-73.

4 Polgreen LA, Carter BL, Polgreen PM, et al. A pharmacist intervention for monitoring and treating hypertension using bidirectional texting: PharmText BP. *Contemp Clin Trials*. 2020;98:106169.

5 Golbus JR, Jeganathan VSE, Stevens R, et al. A Physical Activity and Diet Just-in-Time Adaptive Intervention to Reduce Blood Pressure: The myBPmyLife Study Rationale and Design. *J Am Heart Assoc*. 2024;13(2):e031234.

6 Kao CW, Chen TY, Cheng SM, et al. A Web-Based Self-Titration Program to Control Blood Pressure in Patients With Primary Hypertension: Randomized Controlled Trial. *J Med Internet Res*. 2019;21(12):e15836.

7 Lewinski AA, Patel UD, Diamantidis CJ, et al. Addressing Diabetes and Poorly Controlled Hypertension: Pragmatic mHealth Self-Management Intervention. *J Med Internet Res*. 2019;21(4):e12541.

8 Kerby TJ, Asche SE, Maciosek MV, et al. Adherence to blood pressure telemonitoring in a cluster-randomized clinical trial. *J Clin Hypertens (Greenwich)*. 2012;14(10):668-74.

9 Beger C, Rüegger D, Lenz A, et al. Blood pressure dynamics during home blood pressure monitoring with a digital blood pressure coach-a prospective analysis of individual user data. *Front Cardiovasc Med*. 2023;10:1115987.

10 Ware P, Shah A, Ross HJ, et al. Challenges of Telemonitoring Programs for Complex Chronic Conditions: Randomized Controlled Trial With an Embedded Qualitative Study. *J Med Internet Res*. 2022;24(1):e31754.

11 Margolis KL, Kerby TJ, Asche SE, et al. Design and rationale for Home Blood Pressure Telemonitoring and Case Management to Control Hypertension (HyperLink): a cluster randomized trial. *Contemp Clin Trials*. 2012;33(4):794-803.

12 Gupta A, Chouhdry H, Ellis SD, et al. Design of a pragmatic randomized implementation effectiveness trial testing a health system wide hypertension program for older adults. *Contemp Clin Trials*. 2024;138:107466.

13 Zhang H, Huo X, Ren L, et al. Design and rationale of the Comprehensive intelligent Hypertension managEment SyStem (CHESS) evaluation study: A cluster randomized controlled trial for hypertension management in primary care. *Am Heart J*. 2024;273:90-101.

14 Irizarry T, Allen M, Suffoletto BP, et al. Development and Preliminary Feasibility of an Automated Hypertension Self-Management System. *Am J Med*. 2018;131(9):1125.e1-1125.e8.

15 Maciejewski ML, Bosworth HB, Olsen MK, et al. Do the benefits of participation in a hypertension self-management trial persist after patients resume usual care? *Circ Cardiovasc Qual Outcomes*. 2014;7(2):269-75.

16 Dehmer SP, Maciosek MV, Trower NK, et al. Economic Evaluation of the Home Blood Pressure Telemonitoring and Pharmacist Case Management to Control Hypertension (Hyperlink) Trial. *J Am Coll Clin Pharm*. 2018;1(1):21-30.

17 Castela Forte J, Folkertsma P, Gannamani R, et al. Effect of a Digitally-Enabled, Preventive Health Program on Blood Pressure in an Adult, Dutch General Population Cohort: An Observational Pilot Study. *Int J Environ Res Public Health*. 2022;19(7):4171.

18 Oh SW, Kim KK, Kim SS, et al. Effect of an Integrative Mobile Health Intervention in Patients With Hypertension and Diabetes: Crossover Study. *JMIR Mhealth Uhealth*. 2022;10(1):e27192.

19 Wakefield BJ, Koopman RJ, Keplinger LE, et al. Effect of home telemonitoring on glycemic and blood pressure control in primary care clinic patients with diabetes. *Telemed J E Health*. 2014;20(3):199-205.

20 Thakar V, Kamalakannan S, Prakash V. Effectiveness of m-health technology-enabled physical activity program on physical activity adoption and adherence in people with hypertension in India: A randomized controlled trial protocol. *Chronic Dis Transl Med*. 2023;10(2):92-101.

21 Lee MC, Liu CC, Wang WH, et al. Effectiveness of Mobile Application Disease Self-Management Programme on Mental Health and Self-Management of Patients With Hypertension: A Randomised Controlled Trial. *Int J Nurs Pract*. 2024;30(6):e13312.

22 Vestala H, Bendtsen M, Midlöv P, et al. Effects of an interactive web-based support system via mobile phone on preference-based patient participation in patients living with hypertension - a randomized controlled trial in primary care. *Scand J Prim Health Care*. 2024;42(1):225-233.

23 Liu S, Tanaka R, Barr S, et al. Effects of self-guided e-counseling on health behaviors and blood pressure: Results of a randomized trial. *Patient Educ Couns*. 2020;103(3):635-641.

24 Avegno KS, Roberson KB, Onsomu EO, et al. Evaluating a Telephone and Home Blood Pressure Monitoring Intervention to Improve Blood Pressure Control and Self-Care Behaviors in Adults with Low-Socioeconomic Status. *Int J Environ Res Public Health*. 2023;20(7):5287.

25 Davidson TM, McGillicuddy J, Mueller M, et al. Evaluation of an mHealth Medication Regimen Self-Management Program for African American and Hispanic Uncontrolled Hypertensives. *J Pers Med*. 2015;5(4):389-405.

26 Lokker C, Jezrawi R, Gabizon I, et al. Feasibility of a Web-Based Platform (Trial My App) to Efficiently Conduct Randomized Controlled Trials of mHealth Apps For Patients With Cardiovascular Risk Factors: Protocol For Evaluating an mHealth App for Hypertension. *JMIR Res Protoc*. 2021;10(2):e26155.

27 Meurer WJ, Dome M, Brown D, et al. Feasibility of Emergency Department-initiated, Mobile Health Blood Pressure Intervention: An Exploratory, Randomized Clinical Trial. *Acad Emerg Med*. 2019;26(5):517-527.

28 White WB, Petry NM. Home blood pressure monitoring as an intervention to control hypertension: comment on "Home blood pressure management and improved blood pressure control". *Arch Intern Med*. 2011;171(13):1181-2.

29 Bosworth HB, Olsen MK, McCant F, et al. Hypertension Intervention Nurse Telemedicine Study (HINTS): testing a multifactorial tailored behavioral/educational and a medication management intervention for blood pressure control. *Am Heart J*. 2007;153(6):918-24.

30 Piette JD, Datwani H, Gaudioso S, et al. Hypertension management using mobile technology and home blood pressure monitoring: results of a randomized trial in two low/middle-income countries. *Telemed J E Health*. 2012;18(8):613-20.

31 Chandler J, Sox L, Kellam K, et al. Impact of a Culturally Tailored mHealth Medication Regimen Self-Management Program upon Blood Pressure among Hypertensive Hispanic Adults. *Int J Environ Res Public Health*. 2019;16(7):1226.

32 Jiang Y. Impact of Community Proactive Health Management Application on Electronic Health Literacy and Self-Management of Hypertensive Patients. *Public Health Nurs*. 2024;41(6):1436-1445.

33 Gupta A, Ellis SD, Burkhardt C, et al. Implementing a home-based virtual hypertension programme-a pilot feasibility study. *Fam Pract*. 2023;40(2):414-422.

34 Bengtsson U, Kjellgren K, Hallberg I, et al. Improved Blood Pressure Control Using an Interactive Mobile Phone Support System. *J Clin Hypertens (Greenwich)*. 2016;18(2):101-8.

35 Buis LR, Dawood K, Kadri R, et al. Improving Blood Pressure Among African Americans With Hypertension Using a Mobile Health Approach (the MI-BP App): Protocol for a Randomized Controlled Trial. *JMIR Res Protoc*. 2019;8(1):e12601.

36 Oh SH, Kang JH, Kwon JW. Information and Communications Technology-Based Monitoring Service for Tailored Chronic Disease Management in Primary Care: Cost-Effectiveness Analysis Based on ICT-CM Trial Results. *J Med Internet Res*. 2024;26:e51239.

37 Lakshminarayan K, Murray TA, Westberg SM, et al. Mobile Health Intervention to Close the Guidelines-To-Practice Gap in Hypertension Treatment: Protocol for the mGlide Randomized Controlled Trial. *JMIR Res Protoc*. 2021;10(1):e25424.

38 Varleta P, Acevedo M, Akel C, et al. Mobile phone text messaging improves antihypertensive drug adherence in the community. *J Clin Hypertens (Greenwich)*. 2017;19(12):1276-1284.

39 Jackson GL, Weinberger M, Kirshner MA, et al. Open-label randomized trial of titrated disease management for patients with hypertension: Study design and baseline sample characteristics. *Contemp Clin Trials*. 2016;50:5-15.

40 Wu D, An J, Yu P, et al. Patterns for Patient Engagement with the Hypertension Management and Effects of Electronic Health Care Provider Follow-up on These Patterns: Cluster Analysis. *J Med Internet Res*. 2021;23(9):e25630.

41 Midlöv P, Nilsson PM, Bengtsson U, et al. PERson-centredness in hypertension management using information technology (PERHIT): a protocol for a randomised controlled trial in primary health care. *Blood Press*. 2020;29(3):149-156.

42 Andersson U, Nilsson PM, Kjellgren K, et al. PERson-centredness in Hypertension management using Information Technology: a randomized controlled trial in primary care. *J Hypertens*. 2023;41(2):246-253.

43 Jackson GL, Oddone EZ, Olsen MK, et al. Racial differences in the effect of a telephone-delivered hypertension disease management program. *J Gen Intern Med*. 2012;27(12):1682-9.

44 Bosworth HB, Olsen MK, Grubber JM, et al. Racial differences in two self-management hypertension interventions. *Am J Med*. 2011;124(5):468.e1-8.

45 Meurer WJ, Dinh M, Kidwell KM, et al. Reach out behavioral intervention for hypertension initiated in the emergency department connecting multiple health systems: study protocol for a randomized control trial. *Trials*. 2020;21(1):456.

46 Hermansson-Borrebaeck R, Fors A, Bengtsson U, et al. Self-Efficacy in Hypertension Management Using e-Health Technology: A Randomized Controlled Trial in Primary Care. *J Clin Hypertens (Greenwich)*. 2025;27(1):e14981.

47 Bobrie G, Postel-Vinay N, Delonca J, et al. SETHI Investigators. Self-measurement and self-titration in hypertension: a pilot telemedicine study. *Am J Hypertens*. 2007;20(12):1314-20.

48 Li Y, Maimaitiaili R, Zhang Y, et al. Simplified regimen for the management of hypertension with telemedicine and blood pressure self-monitoring (SIMPLE): study protocol for a randomised controlled trial. *BMJ Open*. 2022;12(3):e049162.

49 Prendergast HM, Petzel-Gimbar R, Kitsiou S, et al. Targeting of uncontrolled hypertension in the emergency department (TOUCHED): Design of a randomized controlled trial. *Contemp Clin Trials*. 2021;102:106283.

50 Buis L, Hirzel L, Dawood RM, et al. Text Messaging to Improve Hypertension Medication Adherence in African Americans From Primary Care and Emergency Department Settings: Results From Two Randomized Feasibility Studies. *JMIR Mhealth Uhealth*. 2017;5(2):e9.

51 Jung H, Lee JE. The impact of community-based eHealth self-management intervention among elderly living alone with hypertension. *J Telemed Telecare*. 2017;23(1):167-173.

52 Yatabe MS, Yatabe J, Asayama K, et al. The rationale and design of reduction of uncontrolled hypertension by Remote Monitoring and Telemedicine (REMOTE) study. *Blood Press*. 2018;27(2):99-105.

53 Lee HY, Kim JY, Na KY, et al. The role of telehealth counselling with mobile self-monitoring on blood pressure reduction among overseas Koreans with high blood pressure in Vietnam. *J Telemed Telecare*. 2019;25(4):241-248.

54 Wong AKC, Bayuo J, Wong FKY, et al. The Synergistic Effect of Nurse Proactive Phone Calls With an mHealth App Program on Sustaining App Usage: 3-Arm Randomized Controlled Trial. *J Med Internet Res*. 2023;25:e43678.

55 Moon EW, Tan NC, Allen JC, et al. The Use of Wireless, Smartphone App-Assisted Home Blood Pressure Monitoring Among Hypertensive Patients in Singapore: Pilot Randomized Controlled Trial. *JMIR Mhealth Uhealth*. 2019;7(5):e13153.

56 Bosworth HB, Olsen MK, Grubber JM, et al. Two self-management interventions to improve hypertension control: a randomized trial. *Ann Intern Med*. 2009;151(10):687-95.

57 Grant S, Hodgkinson J, Schwartz C, et al. Using mHealth for the management of hypertension in UK primary care: an embedded qualitative study of the TASMINH4 randomised controlled trial. *Br J Gen Pract*. 2019;69(686):e612-e620.

58 Fitzpatrick AL, van Pelt M, Heang H, et al. Using Targeted mHealth Messages to Address Hypertension and Diabetes Self-Management in Cambodia: Protocol for a Clustered Randomized Controlled Trial. *JMIR Res Protoc*. 2019;8(3):e11614.

59 Bilger M, Koong AYL, Phoon IKY, et al. Wireless Home Blood Pressure Monitoring System With Automatic Outcome-Based Feedback and Financial Incentives to Improve Blood Pressure in People With Hypertension: Protocol for a Randomized Controlled Trial. *JMIR Res Protoc*. 2021;10(6):e27496.

60 Zhang X, Liao H, Shi D, et al. Cost-effectiveness analysis of different hypertension management strategies in a community setting. *Intern Emerg Med*. 2020;15(2):241-250.

61 Sawitree Visanuyothin. (2017). EFFECTIVENESS OF INTEGRATED HEALTH LITERACY AND SELF-MANAGEMENT MODEL FOR HYPERTENSION CONTROL IN URBAN COMMUNITY, NAKHONRATCHASIMA PROVINCE, THAILAND [ThesisDoctor of Philosophy, Chulalongkorn University]. Chulalongkorn University Intellectual Repository (CUIR). https://doi.org/10.58837/CHULA.THE.2017.499

62 Rubinstein A, Miranda JJ, Beratarrechea A, et al. Effectiveness of an mHealth intervention to improve the cardiometabolic profile of people with prehypertension in low-resource urban settings in Latin America: a randomised controlled trial. *Lancet Diabetes Endocrinol*. 2016;4(1):52-63.

63 Pletcher MJ, Fontil V, Modrow MF, et al. Effectiveness of Standard vs Enhanced Self-measurement of Blood Pressure Paired With a Connected Smartphone Application: A Randomized Clinical Trial. *JAMA Intern Med*. 2022;182(10):1025-1034.

64 Rowland SA, Ramos AK, Trinidad N, et al. mHealth Intervention to Improve Cardiometabolic Health in Rural Hispanic Adults: A Pilot Study. *J Cardiovasc Nurs*. 2022;37(5):439-445.

65 Zare S, Rezaee R, Aslani A, et al. Moving toward community based telehealth services using mhealth for hypertensive patients. *Int J Technol Assess Health Care*. 2019;35(5):379-383.

66 Lokker C, Jezrawi R, Gabizon I, et al. Feasibility of a Web-Based Platform (Trial My App) to Efficiently Conduct Randomized Controlled Trials of mHealth Apps For Patients With Cardiovascular Risk Factors: Protocol For Evaluating an mHealth App for Hypertension. *JMIR Res Protoc*. 2021;10(2):e26155.

67 Persell SD, Karmali KN, Stein N, et al. Design of a randomized controlled trial comparing a mobile phone-based hypertension health coaching application to home blood pressure monitoring alone: The Smart Hypertension Control Study. *Contemp Clin Trials*. 2018;73:92-97.

68 Parati G, Omboni S, Albini F, et al. Home blood pressure telemonitoring improves hypertension control in general practice. The TeleBPCare study. *J Hypertens*. 2009;27(1):198-203.

69 Eldawati E, Said FM, binti Umar NS. The effect of telemonitoring and self-management mobile application on controlling blood pressure among patients with hypertension. *JMIR Preprints*. 2022:44464.

70 Davidson TM, McGillicuddy J, Mueller M, et al. Evaluation of an mHealth Medication Regimen Self-Management Program for African American and Hispanic Uncontrolled Hypertensives. *J Pers Med*. 2015;5(4):389-405.

71 Bilger M, Koong AYL, Phoon IKY, et al. Wireless Home Blood Pressure Monitoring System With Automatic Outcome-Based Feedback and Financial Incentives to Improve Blood Pressure in People With Hypertension: Protocol for a Randomized Controlled Trial. *JMIR Res Protoc*. 2021;10(6):e27496.

72 Hermansson-Borrebaeck R, Andersson U, Jakobsson U, Midlöv P. Beliefs about medications when treating hypertension in primary health care: results from "PERson-centredness in hypertension management using information Technology (PERHIT)". *Blood Press*. 2023;32(1):2226736.

73 McManus RJ, Bray EP, Mant J, et al. Protocol for a randomised controlled trial of telemonitoring and self-management in the control of hypertension: telemonitoring and self-management in hypertension. [ISRCTN17585681]. *BMC Cardiovasc Disord*. 2009;9:6.

74 Bosworth HB, Olsen MK, Dudley T, et al. The Take Control of Your Blood pressure (TCYB) study: study design and methodology. *Contemp Clin Trials*. 2007;28(1):33-47.

75 Aigbonoga D, Adewale B, Igwilo J, et al. Efficacy of short message service (SMS) intervention on medication adherence and knowledge of stroke prevention among clinic attendees at risk of stroke: a randomized controlled trial. *BMC Public Health*. 2025;25(1):1070.

76 Ashoorkhani M, Bozorgi A, Majdzadeh R, et al. Comparing the effectiveness of the BPMAP (Blood Pressure Management Application) and usual care in self-management of primary hypertension and adherence to treatment in patients aged 30-60 years: study protocol for a randomized controlled trial. *Trials*. 2016;17(1):511.

77 Band R, Morton K, Stuart B, et al. Home and Online Management and Evaluation of Blood Pressure (HOME BP) digital intervention for self-management of uncontrolled, essential hypertension: a protocol for the randomised controlled HOME BP trial. *BMJ Open*. 2016;6(11):e012684.

78 Bernocchi P, Scalvini S, Bertacchini F, et al. Home based telemedicine intervention for patients with uncontrolled hypertension--a real life non-randomized study. *BMC Med Inform Decis Mak*. 2014;14:52.

79 Bozorgi A, Hosseini H, Eftekhar H, et al. The effect of the mobile "blood pressure management application" on hypertension self-management enhancement: a randomized controlled trial. *Trials*. 2021;22(1):413.

80 Debon R, Bellei EA, Biduski D, et al. Effects of using a mobile health application on the health conditions of patients with arterial hypertension: A pilot trial in the context of Brazil's Family Health Strategy. *Sci Rep*. 2020;10(1):6009.

81 Erden Y, Yıldız GN, Çiftçi B, et al. The effect of self-management program with tele-nursing based on the Roper-Logan-Tierney model on self-care of hypertensive patients: a randomized controlled trial. *BMC Nurs*. 2025;24(1):313.

82 Franssen M, Farmer A, Grant S, et al. Telemonitoring and/or self-monitoring of blood pressure in hypertension (TASMINH4): protocol for a randomised controlled trial. *BMC Cardiovasc Disord*. 2017;17(1):58.

83 Gazit T, Gutman M, Beatty AL. Assessment of Hypertension Control Among Adults Participating in a Mobile Technology Blood Pressure Self-management Program. *JAMA Netw Open*. 2021;4(10):e2127008.

84 Hammersley V, Parker R, Paterson M, et al. Telemonitoring at scale for hypertension in primary care: An implementation study. *PLoS Med*. 2020;17(6):e1003124.

85 Hartch CE, Dietrich MS, Lancaster BJ, Stolldorf DP, Mulvaney SA. Effects of a medication adherence app among medically underserved adults with chronic illness: a randomized controlled trial. *J Behav Med*. 2024;47(3):389-404.

86 Ionov MV, Zhukova OV, Zvartau NE, et al. [Assessment of the clinical efficacy of telemonitoring and distant counseling in patients with uncontrolled hypertension]. *Ter Arkh*. 2020;92(1):49-55.

87 Kwan YH, Yoon S, Tai BC, et al. Empowering patients with comorbid diabetes and hypertension through a multi-component intervention of mobile app, health coaching and shared decision-making: Protocol for an effectiveness-implementation of randomised controlled trial. *PLoS One*. 2024;19(2):e0296338.

88 Li T, Ding W, Li X, Lin A. Mobile health technology (WeChat) for the hierarchical management of community hypertension: protocol for a cluster randomized controlled trial. *Patient Prefer Adherence*. 2019;13:1339-1352.

89 Margolis KL, Crain AL, Green BB, et al. Comparison of explanatory and pragmatic design choices in a cluster-randomized hypertension trial: effects on enrollment, participant characteristics, and adherence. *Trials*. 2022;23(1):673.

90 McManus RJ, Bray EP, Mant J, et al. Protocol for a randomised controlled trial of telemonitoring and self-management in the control of hypertension: telemonitoring and self-management in hypertension. [ISRCTN17585681]. *BMC Cardiovasc Disord*. 2009;9:6.

91 Parati G, Omboni S, Compare A, et al. Blood pressure control and treatment adherence in hypertensive patients with metabolic syndrome: protocol of a randomized controlled study based on home blood pressure telemonitoring vs. conventional management and assessment of psychological determinants of adherence (TELEBPMET Study). *Trials*. 2013;14:22.

92 Pati S, Menon J, Rehman T, et al. Developing and assessing the "MultiLife" intervention: a mobile health-based lifestyle toolkit for cardiometabolic multimorbidity in diabetes and hypertension management - a type 1 hybrid effectiveness-implementation trial protocol. *BMC Public Health*. 2025;25(1):3.

93 Schlichtiger J, Strüven A, Massberg S, et al. Evaluation of a digital therapy programme for the treatment of primary arterial hypertension: eXPLORE - study protocol for a fully decentralised randomised controlled feasibility study. *BMJ Open*. 2024;14(9):e081347.

94 Shen X, Xiao S, Liu R, et al. Personalized hypertension management based on serial assessment and telemedicine (PHMA): a cluster randomize controlled trial protocol in Anhui, China. *BMC Cardiovasc Disord*. 2021;21(1):135.

95 Taher M, Yule C, Bonaparte H, et al. Telehealth versus self-directed lifestyle intervention to promote healthy blood pressure: a protocol for a randomised controlled trial. *BMJ Open*. 2021;11(3):e044292.

96 Wang Z, Li C, Huang W, et al. Effectiveness of a pathway-driven eHealth-based integrated care model (PEICM) for community-based hypertension management in China: study protocol for a randomized controlled trial. *Trials*. 2021;22(1):81.

**Supplementary Figure1. Forest Plots for the Various Outcome Measures.**

**
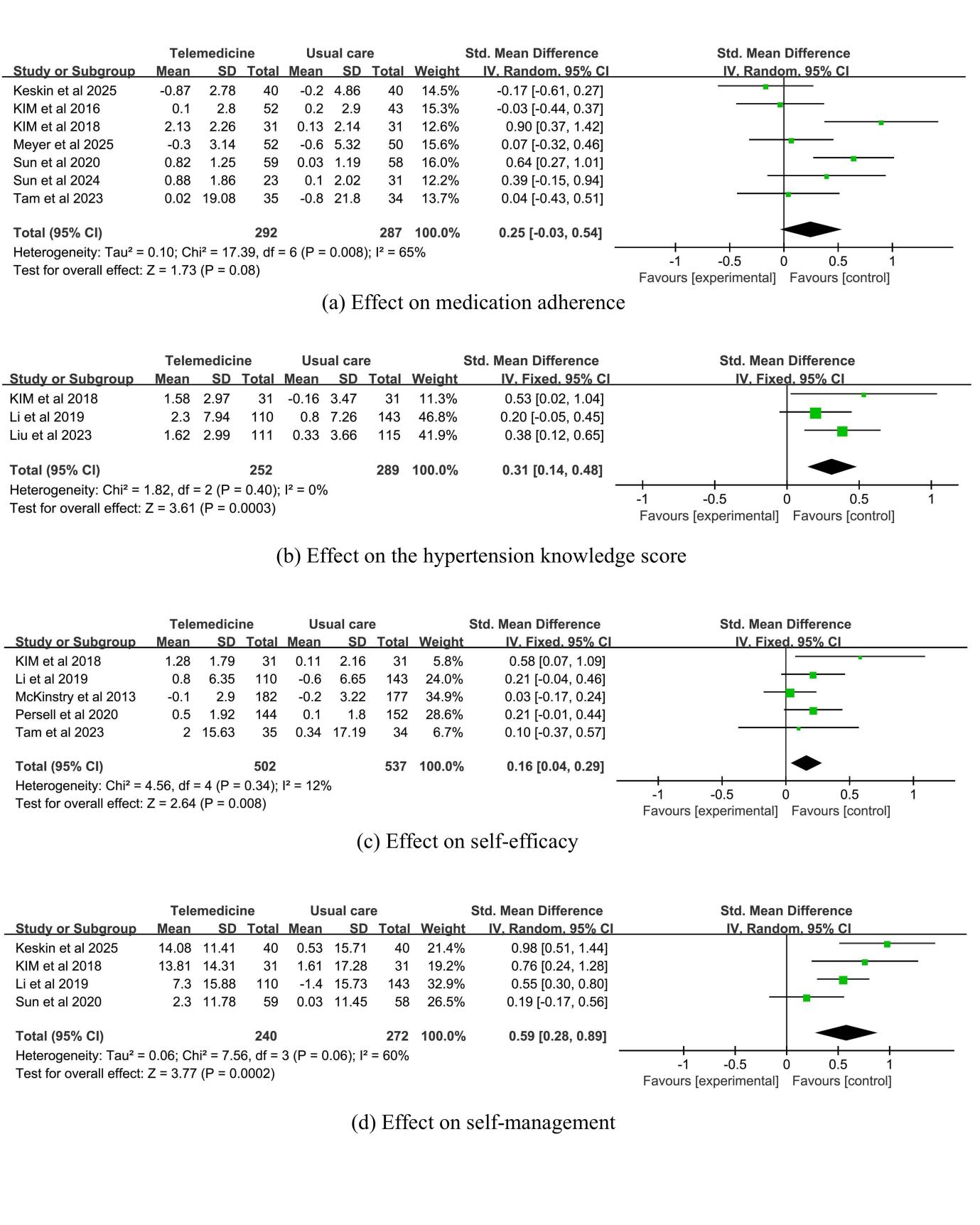
**

**
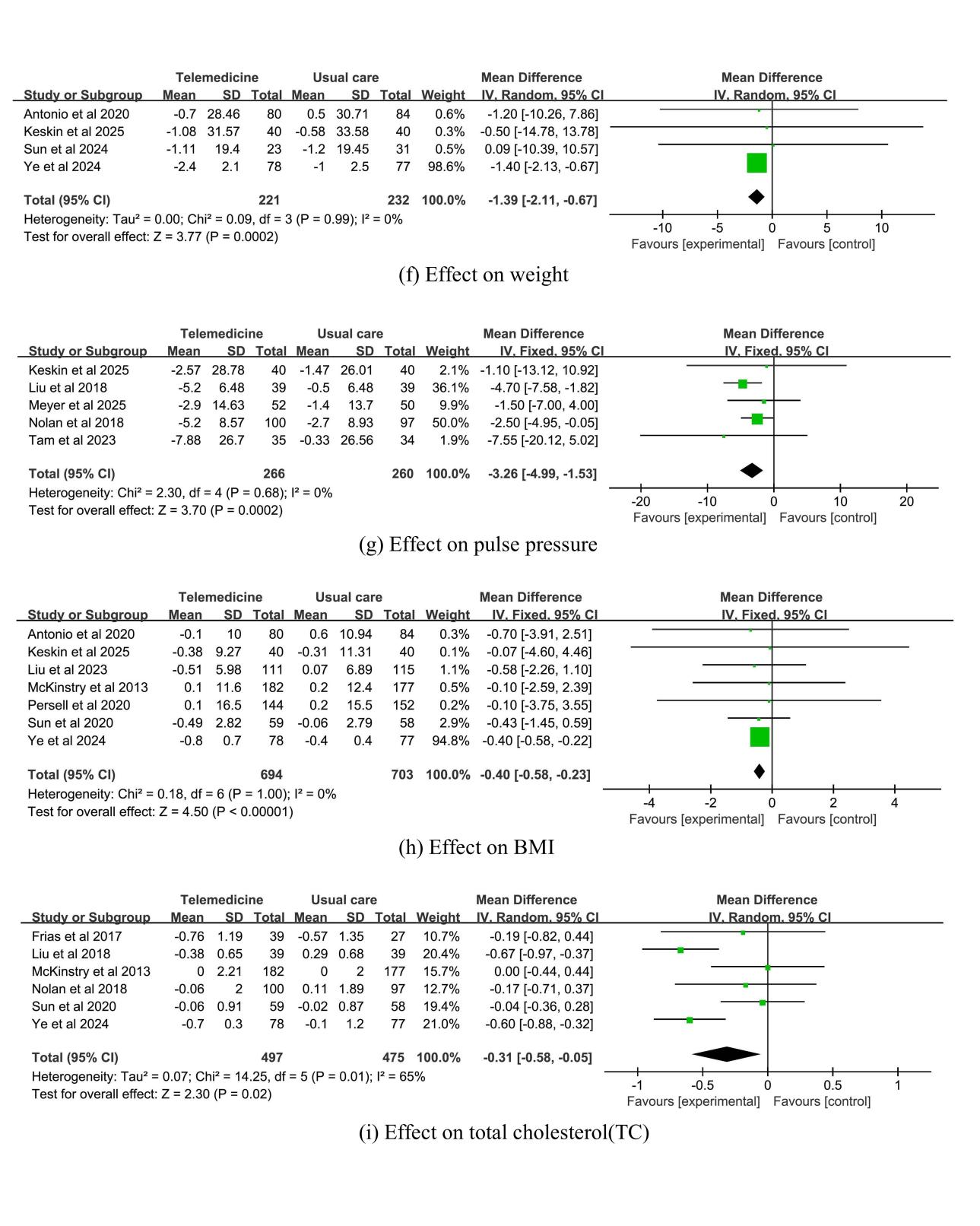

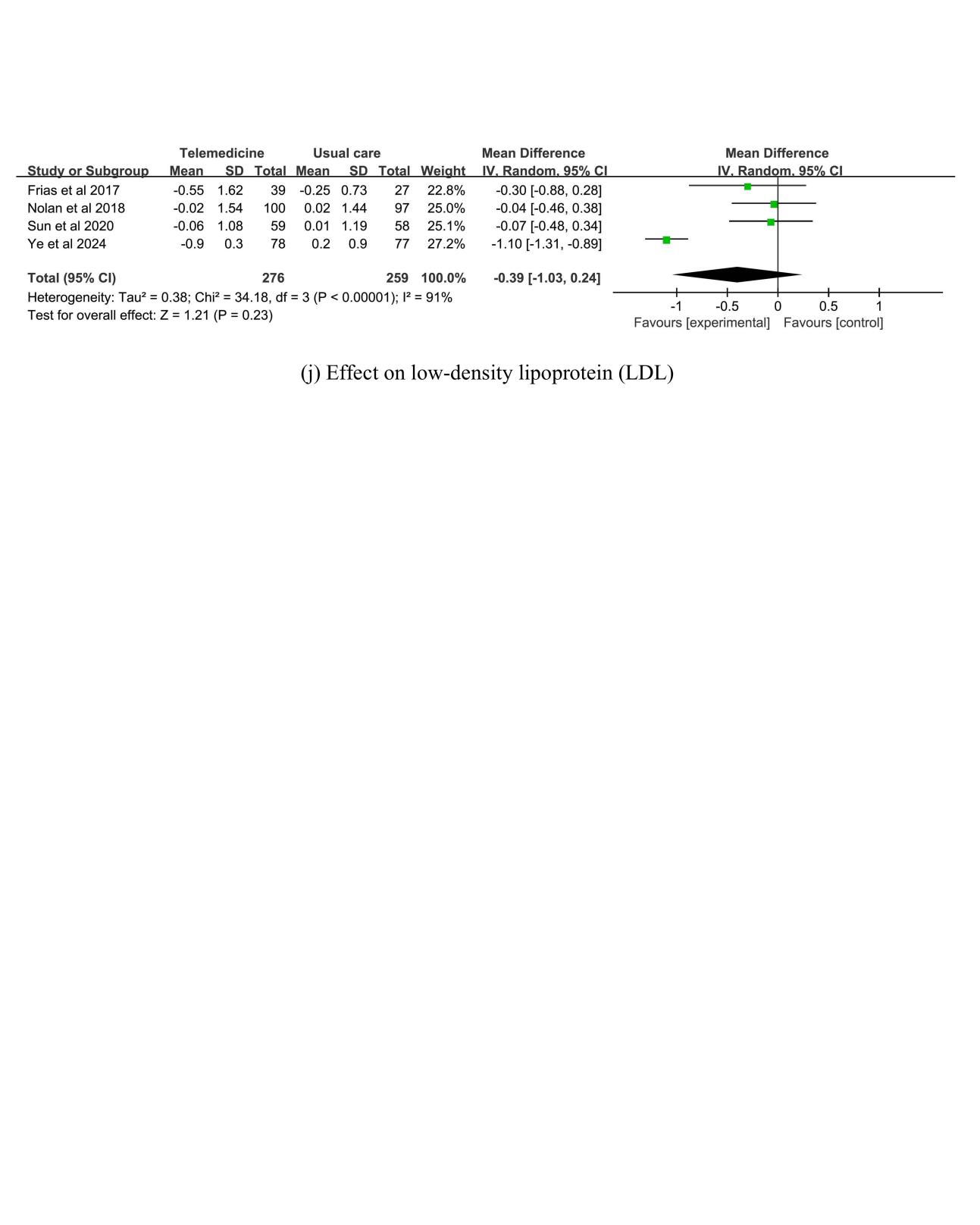
**

**Supplementary Figure2. Funnel Plots for Assessment of Publication Bias Across All Outcome Measures**

**
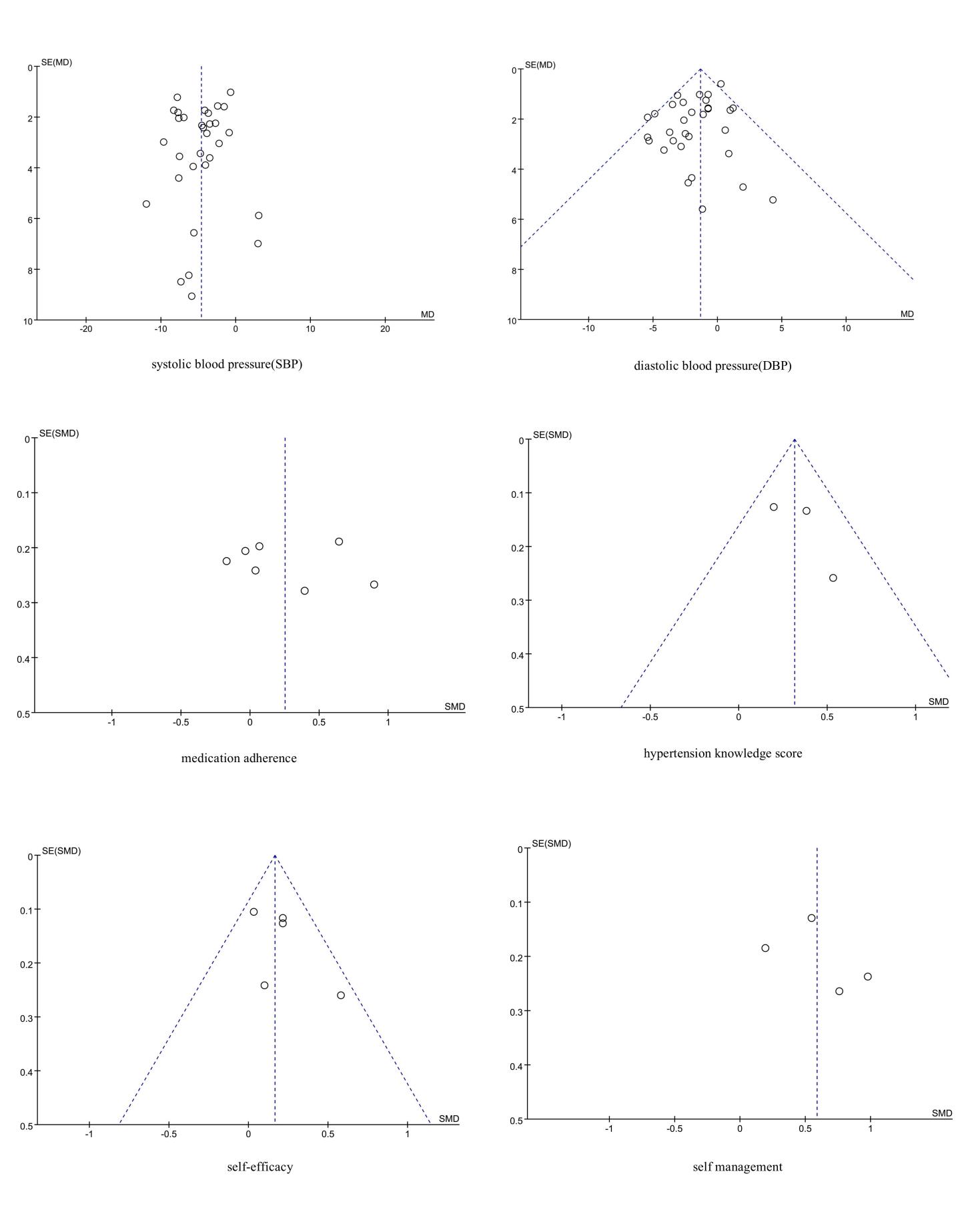
**

**
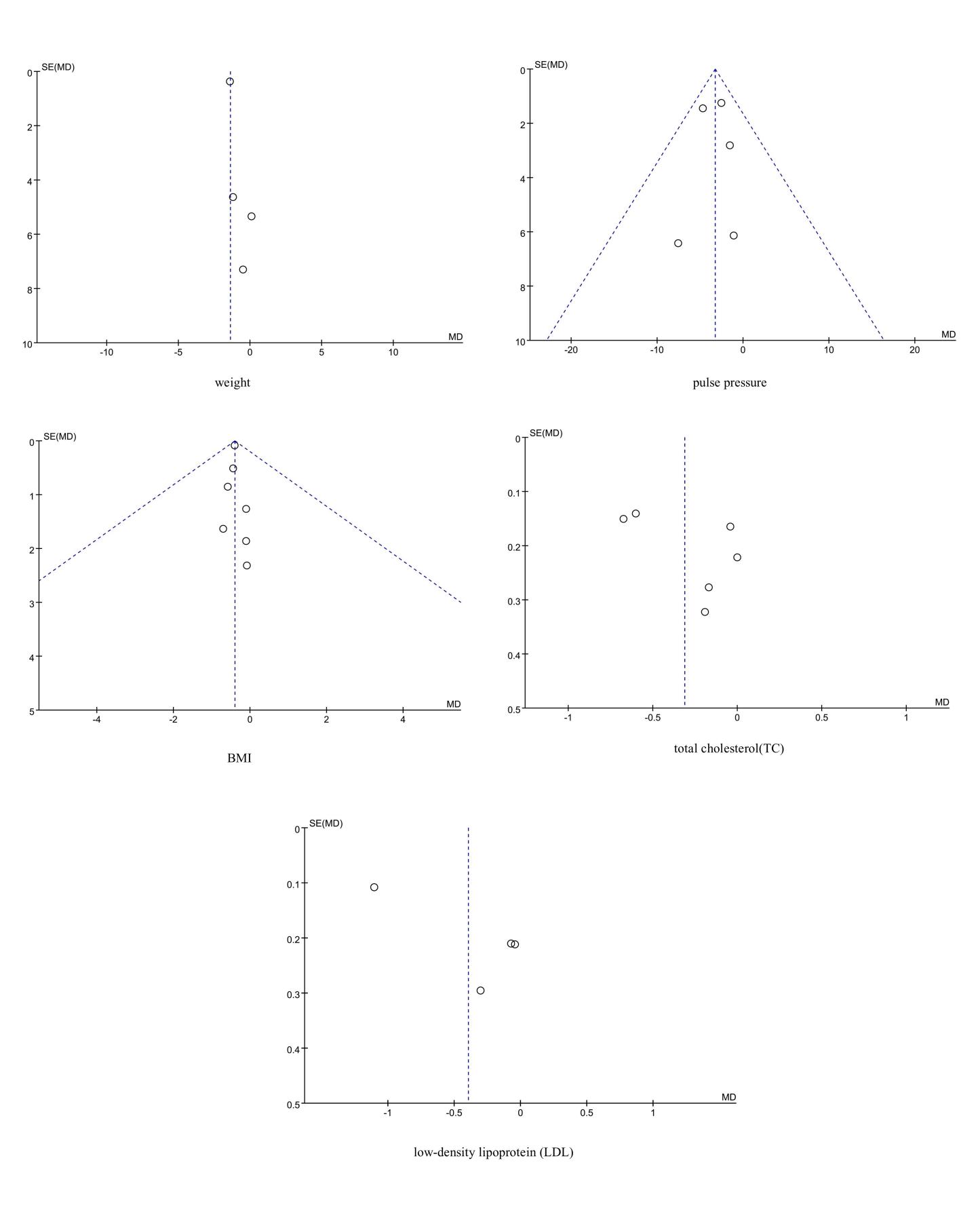
**

**Supplementary Figure3. Funnel Plots Assessing Publication Bias for Subgroup Analyses of Systolic and Diastolic Blood Pressure**

**
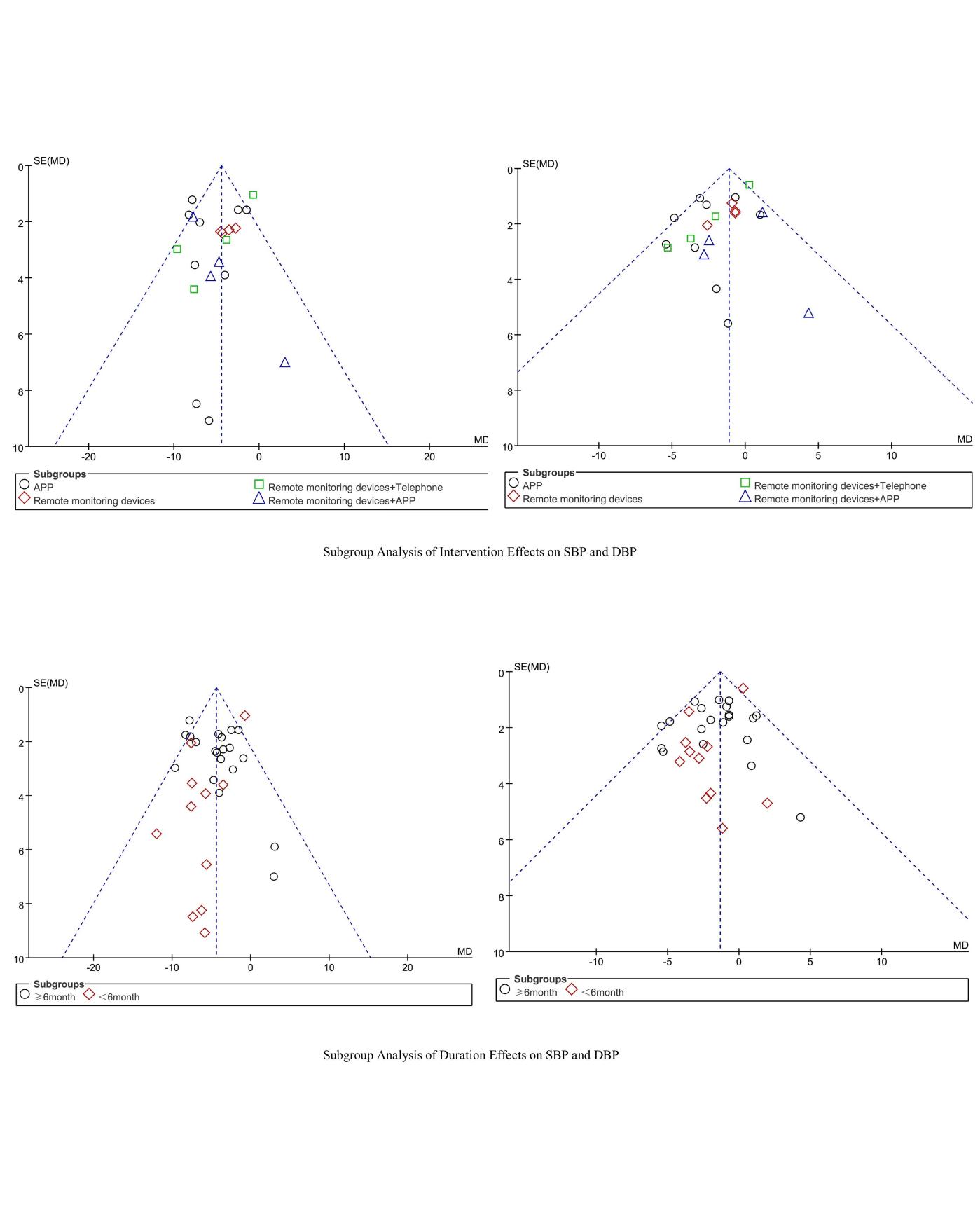
**

**
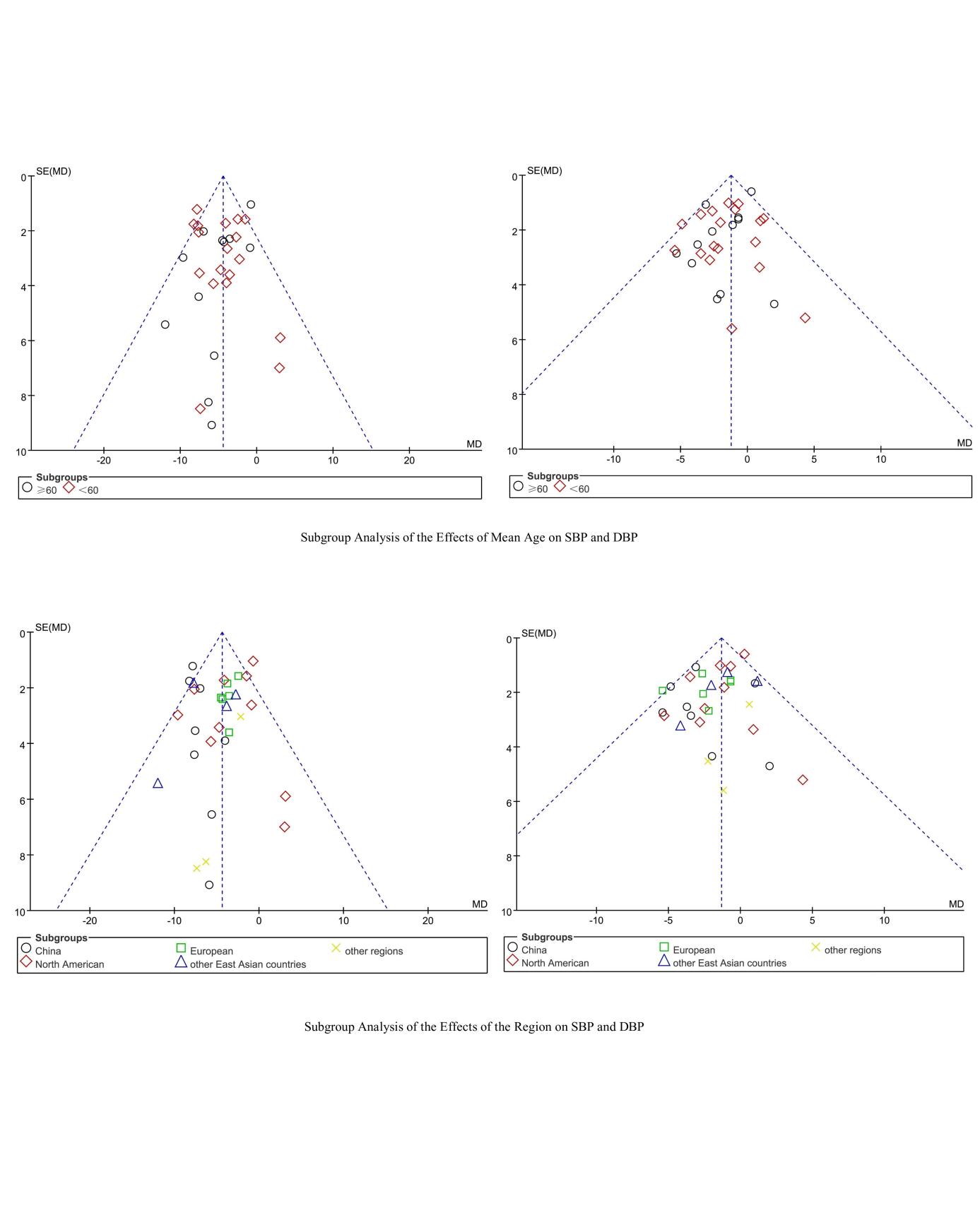
**

**
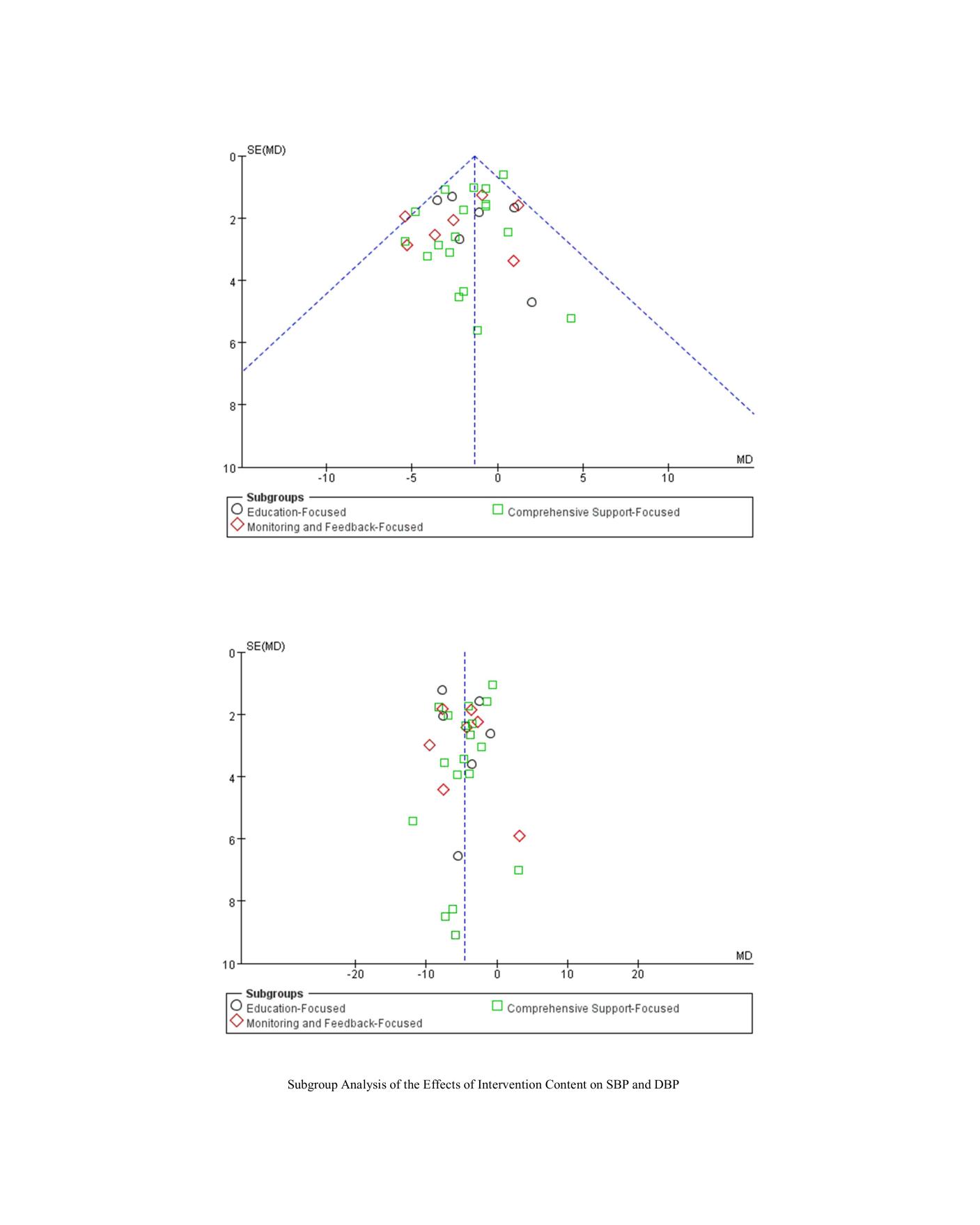
**
